# Associations between obstructive sleep apnoea and the development and severity of retinal vein occlusion

**DOI:** 10.1038/s41433-025-03900-4

**Published:** 2025-07-16

**Authors:** Hejin Jeong, Jacqueline K. Shaia, David C. Kaelber, Katherine E. Talcott, Rishi P. Singh

**Affiliations:** 1 Case Western Reserve University School of Medicine, Cleveland, OH USA; 2 Center for Ophthalmic Bioinformatics, Cole Eye Institute, Cleveland Clinic, Cleveland, OH USA; 3 Center for Clinical Informatics Research and Education, MetroHealth System and the Departments of Internal Medicine, Pediatrics, and Population and Quantitative Health Sciences, Case Western Reserve University School of Medicine, Cleveland, OH USA; 4 Cleveland Clinic Martin Health, Cleveland Clinic Florida, Stuart, FL USA

**Keywords:** Retinal diseases, Epidemiology, Obesity, Risk factors, Respiratory tract diseases

## Abstract

**Background/Objectives:**

Emerging research suggests obstructive sleep apnoea (OSA) as a potential risk factor for retinal vein occlusion (RVO), but the impact of sex, race, and ethnicity, and the role of OSA in RVO progression, remains unclear. This study explored demographic differences in the association between OSA and RVO and compared the severity of RVO in patients with and without OSA.

**Subjects/Methods:**

This retrospective cohort study analysed aggregated, de-identified electronic health record data of US patients. Adults who received ophthalmological services were grouped by baseline RVO status (RVO-naïve and preexisting RVO). The RVO-naïve group was further stratified by sex, race, and ethnicity. Patients with and without OSA were compared within each group to evaluate the risk ratio for primary outcomes: new RVO diagnoses in RVO-naïve individuals and RVO complications or invasive treatments in those with preexisting RVO.

**Results:**

Among RVO-naïve adults, OSA was associated with an increased risk of RVO in females (*n* = 148,036, RR = 1.28, CI = 1.14–1.45), males (*n* = 134,348, RR = 1.35, CI = 1.19–1.52), non-Hispanic White (*n* = 146,124, RR = 1.32, CI = 1.17–1.49), Hispanic/Latino (*n* = 30,898, RR = 1.77, CI = 1.30–2.40) patients. A marginally increased risk was seen in Black patients (*n* = 57,798, RR = 1.26, CI = 1.05–1.50), but not in Asian patients (*n* = 6860, RR = 1.21, CI = 0.71–2.07). Among 5264 adults with preexisting RVO, those with OSA had higher rates of macular oedema (RR = 3.70, CI = 3.17–4.31), vitreous haemorrhage (RR = 2.29, CI = 1.64–3.20), neovascularization (RR = 2.22, CI = 1.69–2.91), and photocoagulation (RR = 1.73, CI = 1.29–2.33), but not vitrectomy (RR = 1.13, CI = 0.74–1.72).

**Conclusions:**

OSA is associated with an increased risk of RVO among various populations, especially among Hispanic/Latino. Among patients with preexisting RVO, OSA is associated with indicators of more severe RVO.

## Introduction

Obstructive sleep apnoea (OSA) is the most common sleep-breathing disorder that results in chronic nocturnal hypoxia and hypertension [1], and is estimated to affect over 20% of the United States (US) and the global population [2]. Emerging studies have indicated that OSA may also increase the risks of developing retinal vein occlusion (RVO) [3–5], possibly due to hypoxia- and hypercapnia-induced vasodilation that leads to blood stasis. This may trigger RVO, especially in the state of increased oxidative stress, inflammation, and increased respiratory effort that occurs in OSA [4, 6–8].

However, the results of these studies have limited generalizability, as they largely consist of small single-institution studies, with only one national population-wide study [3] that was conducted in a Taiwanese population. Furthermore, even though OSA disproportionately affects males and minority populations [1, 9, 10], the impact of race, ethnicity, and sex among patients with OSA has rarely been investigated in the context of RVO. Finally, at present, research on how OSA may impact the severity of RVO is lacking, despite RVO’s potential to cause significant complications such as macular oedema, neovascularization, and vision loss. Given such gaps in current knowledge, this study aimed to analyse a large aggregate platform composed of US data to better characterize any race-, ethnicity-, and sex-related differences in the relationships between OSA and RVO development, and to further investigate potential associations between OSA the risks of the progression of RVO.

## Methods

This retrospective cohort investigation utilized the US Collaborative Network of the TriNetX Platform, a health research network consolidating de-identified electronic health records (EHR) of over 110 million patients from more than 60 US healthcare organizations. These institutions include both hospital and outpatient care facilities and both insured and uninsured patients, enabling a wider range of patient demographics to be represented. This retrospective study is exempt from informed consent. The data reviewed is a secondary analysis of existing data, does not involve intervention or interaction with human subjects, and is de-identified per the de-identification standard defined in Section §164.514(a) of the HIPAA Privacy Rule. The process by which the data is deidentified is attested to through a formal determination by a qualified expert as defined in Section §164.514(b) [1] of the HIPAA Privacy Rule. This formal determination by a qualified expert was refreshed in December 2020. This study follows the STROBE reporting guidelines for cohort studies.

Queries used for the data analysis were based on the International Statistical Classification of Diseases and Related Health Problems, Tenth Revision (ICD-10), Anatomical Therapeutic Chemical (ATC), RxNorm, Logical Observation Identifier Names and Codes (LOINC), and Current Procedural Terminology (CPT) codes. Any diagnoses that occurred before the year 2015 and were recorded in ICD-9 codes were converted to the corresponding ICD-10 codes based on the Centres for Medicare and Medicaid Services General Equivalence Mappings. The relevant codes used can be found in eTables 1–3. Data were accessed on July 28^th^, 2024.

This study compared patients with and without any OSA ICD code to achieve two primary objectives: (1) to perform sex- and race/ethnicity-stratified analysis on the risks of being diagnosed with RVO among patients with no recorded history of RVO ICD diagnosis codes (“RVO-naïve”), and (2) to determine the relationship between OSA ICD code and the risk of clinically significant progression of RVO among patients with a preexisting ICD-10 diagnosis code for RVO (“preexisting-RVO” cohort).

This study included patients aged ≥18 years who had a recorded ICD-10 diagnosis or service code for ophthalmological services or procedures (eTable 1). Patients were excluded if they had a record of the outcome before the index date, which was defined as the first date when all inclusion criteria were satisfied (eTable 1). Then, two cohorts—“OSA” and “control” cohorts—were created from the pool of patients who satisfied these inclusion criteria. The OSA cohort consisted of those with an ICD diagnosis code for OSA (G47.33) and a record of undergoing at least one polysomnography (CPT 1013314), while the control cohort consisted of those without any sleep apnoea (G47.3, G47.9) ICD diagnoses, including OSA (eTable 1).

All analyses in this study were performed using the following general procedure: (1) identifying the OSA and control cohorts (2) matching the OSA cohort to the control cohort in each subgroup via propensity score matching (PSM) analysis; (3) comparing cohorts to evaluate the risk ratios (RR) of receiving a diagnosis code for the outcome of interest.

### Investigating the associations between OSA and the risks of being diagnosed with RVO in various race, ethnicity, and sex subgroups

To achieve the first objective of this study, patients who satisfied the inclusion criteria were first stratified into subgroups based on sex—male and female—and race/ethnicity—non-Hispanic and non-Latino White (NHW), Hispanic or Latino (any race) (H/L), Black, and Asian (eTable 1).

PSM was performed to match the OSA and the control cohorts within each subgroup on baseline age, sex, ethnicity, race, tobacco and alcohol use, body mass index, haemoglobin A1c, history of systemic comorbidities, other common sleep disorders, continuous positive airway pressure (CPAP) therapy, glaucoma, diabetic ocular diseases, and age-related macular degeneration (AMD), and the use of hypnotic agents, medications that modulate thrombotic risks, and antihypertensives (eTable 2).

**Fig. 1:**
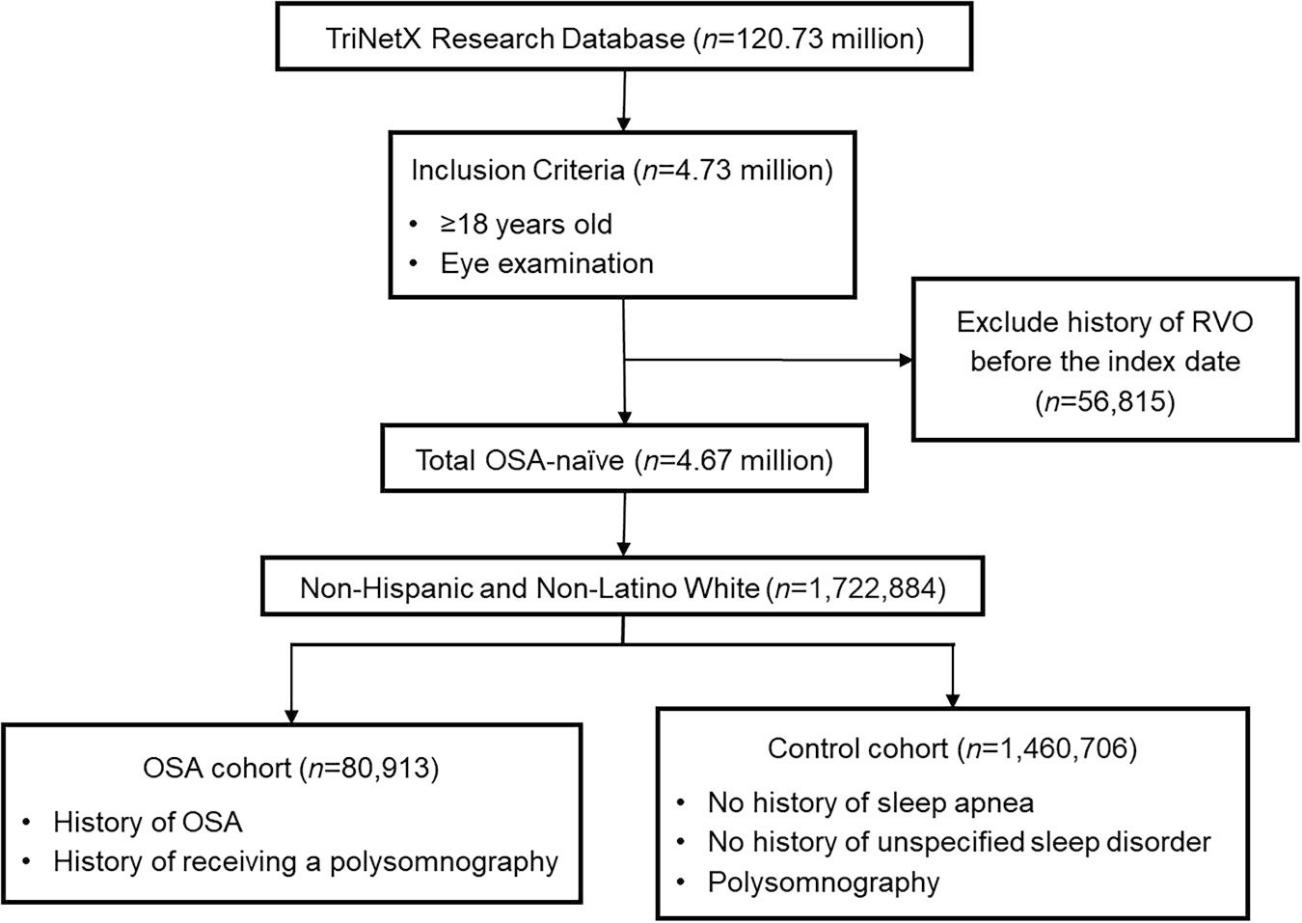
CONSORT diagram sample for the Non-Hispanic and Non-Latino White Population.

Following PSM, the RVO-naive cohorts with and without any OSA ICD code were compared to evaluating the RR of receiving a new ICD diagnosis code for any RVO, which was defined as central RVO (CRVO) or branch RVO (BRVO) (eTable 1). To assess any differences in the relationships between OSA and different types of RVO, the risks of receiving a new ICD code for CRVO and BRVO were additionally analysed among CRVO-naïve and BRVO-naïve patients, respectively. An example CONSORT diagram and summary of the inclusion and exclusion criteria for the analysis of NHW patients are shown in Fig. 1 and eTable 3, respectively.

### Investigating the associations between OSA and the risks of RVO progression

For the analysis of the risks of adverse progression of RVO, only patients with a record of any preexisting ICD code for RVO were selected from the initial group of patients who satisfied the general inclusion criteria outlined above. Patients were again divided into the OSA and the control cohorts. In addition to the covariates that were used in the PSM analysis on RVO-naïve patients, this analysis also matched the cohorts on which type of RVO—CRVO or BRVO—the patients had at baseline (eTable 2).

Adverse progression of RVO was defined as having code for a complication of RVO or an invasive treatment procedure. Complications of RVO included macular oedema (MO), vitreous haemorrhage (VH), and neovascularization of the eye (NV); invasive treatments included pars plana vitrectomy (PPV), pan-retinal photocoagulation (PRP). The number of intravitreal injections, such as ophthalmic anti-vascular endothelial growth factor (VEGF) agents or corticosteroids (eTable 1), was also compared.

As with the analysis on RVO-naïve patients, patients who had a record of the outcome before the index date were excluded from the final analysis, and the RR of having the adverse clinical event was evaluated. For the number of intravitreal injections only, the student t-test was used to evaluate RVO progression instead of the RR, as the number of intravitreal injections is a quantitative variable.

### Sensitivity analysis

While we chose broad inclusion criteria for the primary analysis on the progression of RVO to optimize the statistical power and patient diversity, it is important to recognize that the outcome measures used in this analysis may occur due to other ocular conditions besides RVO. For instance, severe or proliferative diabetic retinopathy (DR) may cause ME, VH, and NV, and their complications may require PPV, PRP, and anti-VEGF treatment. As such, a sensitivity analysis was performed by excluding patients who had any of the outcome measures if they also had a record of any ICD code for any of the following confounding ocular conditions within one year before the occurrence of the outcome: severe non-proliferative or proliferative DR, exudative AMD, macular pucker, cyst, hole, or pseudohole, retinal cysts, glaucoma, endophthalmitis, degenerative myopia, complications of ophthalmic procedures, ocular trauma, and cataract surgery (eTable 1).

### Statistical analysis

All statistical analyses were performed using the built-in analytics tool provided by the database. PSM analysis utilized a one-to-one greedy matching algorithm with a calliper of 0.25 pooled standard deviations. Cohorts were considered to be adequately balanced if the absolute value of the standardized mean difference (SMD) for each covariate was 0.1 or less. The RR of receiving a code for the outcome and the corresponding 95% confidence intervals (CI) were calculated via logistic regression. To account for multiple sampling, statistical significance was defined as a 95% CI of ≤0.9 and ≥1.1, or a *p* value of ≤0.01.

## Results

The analysis contained 3,279,582 RVO-naïve patients (mean age: 50.2 ± 21.2, 56.9% female, 50.6% NHW, 10.8% H/L, 13.9% Black, 4.5% Asian) and 19,918 RVO patients (mean age: 68.3 ± 14.5, 53.0% female, 38.8% CRVO) who fulfilled the inclusion criteria. After PSM, all analyses achieved adequate balance (SMD < 0.1) on all covariates (eTable 4).

### Risks of being diagnosed with RVO

The results of the comparative analyses among RVO-naïve patients are summarized in Table 1.

**Table 1:** Age- and race-stratified risk of retinal vein occlusion diagnosis in patients with no prior history of retinal vein occlusion.

Patient Group	Outcome	OSA		Control (No OSA)		Risk Ratio	95% Confidence Interval
		Event / Total		Event / Total			
Male	RVO	601/67,174	(0.89%)	447/67,174	(0.67%)	**1.35**	**1.19–1.52**
	CRVO	299/67,174	(0.45%)	235/67,174	(0.35%)	1.27	1.07–1.51
	BRVO	384/67,174	(0.57%)	261/67,174	(0.39%)	**1.47**	**1.26–1.72**
Female	RVO	594/74,018	(0.80%)	463/74,018	(0.63%)	**1.28**	**1.14–1.45**
	CRVO	283/74,018	(0.38%)	228/74,018	(0.31%)	1.24	1.04–1.48
	BRVO	401/74,018	(0.54%)	291/74,018	(0.39%)	**1.38**	**1.19–1.60**
Non-Hispanic and Non-Latino White	RVO	602/73,062	(0.82%)	456/73,062	(0.62%)	**1.32**	**1.17–1.49**
	CRVO	270/73,062	(0.37%)	215/73,062	(0.29%)	1.26	1.05–1.50
	BRVO	416/73,062	(0.57%)	293/73,062	(0.40%)	**1.42**	**1.22–1.65**
Hispanic or Latino	RVO	113/15,449	(0.73%)	64/15,449	(0.41%)	**1.77**	**1.30–2.40**
	CRVO	57/15,449	(0.37%)	38/15,449	(0.25%)	1.50	1.00–2.26
	BRVO	69/15,449	(0.45%)	37/15,449	(0.24%)	**1.87**	**1.25–2.78**
Black	RVO	279/28,899	(0.97%)	222/28,899	(0.77%)	1.26	1.05–1.50
	CRVO	145/28,899	(0.50%)	124/28,899	(0.43%)	1.17	0.92–1.49
	BRVO	178/28,899	(0.62%)	128/28,899	(0.44%)	**1.39**	**1.11–1.74**
Asian	RVO	29/3430	(0.85%)	24/3430	(0.70%)	1.21	0.71–2.07
	CRVO	11/3430	(0.32%)	11/3430	(0.32%)	1.00	0.43–2.30
	BRVO	20/3430	(0.58%)	15/3430	(0.44%)	1.33	0.68–2.60

*RVO* retinal vein occlusion, *CRVO* central retinal vein occlusion, *BRVO* branch retinal vein occlusion, *OSA* obstructive sleep apnoea.

Regardless of sex, RVO-naïve patients with an OSA ICD code had a higher risk of having a new ICD code of any RVO (males: RR = 1.35, CI = 1.19–1.52; females: RR = 1.28, CI = 1.14–1.45) and BRVO (males: RR = 1.47, CI = 1.26–1.72; females: RR = 1.38, CI = 1.19–1.60) compared to those without any recorded instance of OSA ICD code. The risk of having a new ICD code of CRVO did not differ between the OSA and the control cohorts in either sex (males: RR = 1.27, CI = 1.07–1.51; females: RR = 1.24, CI = 1.04–1.48).

The race- and ethnicity-stratified analysis revealed that patients in the OSA cohort had a higher risk of being newly diagnosed with RVO and BRVO compared to controls, with the highest risk observed in the H/L population (any RVO: RR = 1.77, CI = 1.30–2.40; BRVO: RR = 1.87, CI = 1.25–2.78), followed by NHW patients (any RVO: RR = 1.32, CI = 1.17–1.49; BRVO: RR = 1.42, CI = 1.22–1.65), and a marginally significant increase in risk among Black patients (any RVO: RR = 1.26, CI = 1.05–1.50; BRVO: RR = 1.39, CI = 1.11–1.74). In contrast, no such association was observed within the Asian population (any RVO: RR = 1.21, CI = 0.71–2.07; BRVO: RR = 1.33, CI = 0.68–2.60).

As in the sex-stratified analysis, the risk of receiving a new ICD code for CRVO did not significantly differ between the OSA and the control cohorts in any racial or ethnic group (NHW: RR = 1.26, CI = 1.05–1.50; H/L: RR = 1.50, CI = 1.00–2.26; Black: RR = 1.17, CI = 0.92–1.49; Asian: RR = 1.00, CI = 0.43–2.30).

### Risks of RVO progression

Among patients with a baseline ICD code of RVO, a higher proportion of patients in the OSA cohort had a new ICD code of MO (RR = 3.70, CI = 3.17–4.31), VH (RR = 2.29, CI = 1.64–3.20), or NV (RR = 2.22, CI = 1.69–2.91), and had a record of undergoing PRP (RR = 1.73, CI = 1.29–2.33) for treatment compared to controls (Table 2A). Similarly, the number of injections administered was significantly higher in the OSA cohort than in the control cohort (*t* = 8.062, *p* < 0.0001) (Table 2B). In contrast, no such difference was observed for the rate of PPV treatment between the two cohorts (RR = 1.13, CI = 0.74–1.72) (Table 2A). These findings were consistent in the sensitivity analyses (Table 2A, B).

**Table 2:** A: Risk of adverse progression of retinal vein occlusion severity in patients with a preexisting history of retinal vein occlusion. B: Number of intravitreal injections that patients with a preexisting history of retinal vein occlusion received.

A							
Analysis	Outcome	OSA		Control (No OSA)		Risk ratio	95% Confidence interval
		Event / Total		Event / Total			
Primary analysis	Vitrectomy	45/2,632	(1.71%)	40/2632	(1.52%)	1.13	0.74–1.72
	Photocoagulation	116/2632	(4.41%)	67/2632	(2.55%)	**1.73**	**1.29–2.33**
	Macular oedema	680/2632	(25.84%)	184/2632	(6.99%)	**3.70**	**3.17–4.31**
	Vitreous haemorrhage	110/2632	(4.18%)	48/2632	(1.82%)	**2.29**	**1.64–3.20**
	Neovascularization	162/2632	(6.16%)	73/2632	(2.77%)	**2.22**	**1.69–2.91**
Sensitivity analysis^a^	Vitrectomy	18/896	(2.01%)	<10^b^/896	(1.12%)	1.80	0.84–3.88
	Photocoagulation	31/896	(3.46%)	<10^b^/896	(1.12%)	**3.10**	**1.53–6.29**
	Macular oedema	288/896	(32.14%)	11/896	(1.23%)	**26.18**	**14.44–47.47**
	Vitreous haemorrhage	37/896	(4.13%)	<10^b^/896	(1.12%)	**3.70**	**1.85–7.39**
	Neovascularization	58/896	(6.47%)	<10^b^/896	(1.12%)	**5.80**	**2.98–11.28**

B								
Analysis	OSA			Control			t	*p*value
	*n*	Mean	SD	*n*	Mean	SD		
Primary analysis	443	1.16	4.13	304	0.41	1.59	8.06	<0.0001
Sensitivity analysis^c^	145	1.31	4.71	41	0.08	0.42	7.57	<0.0001

*OSA* obstructive sleep apnoea, *SD* standard deviation.

^a^The sensitivity analysis was performed by excluding patients who had ocular conditions at baseline.

^b^To protect patient privacy, TriNetX reports any counts between 1 and 10 as 10. This may impact results, particularly for small cohorts and infrequent outcomes.

^c^The sensitivity analysis was performed by excluding patients who had ocular conditions within a year of the occurance of the analysed outcome.

## Discussion

In this study comparing a large cohort of patients with and without a recorded ICD code for OSA among various racial/ethnic and sex subgroups, OSA was found to be associated with an increased risk of having a new ICD code of RVO in both males and females, and NHW, Black, and H/L population. These findings are consistent with those of existing studies [3–5, 11] that have investigated the prevalence of RVO among patients with OSA in the general population. Moreover, even among those with preexisting ICD codes for RVO, those with OSA had an increased risk of being diagnosed with vision-threatening complications and of undergoing invasive treatment procedures.

Although the current literature lacks specific data on how sex influences the relationship between OSA and RVO, epidemiological studies consistently show that males are at a significantly higher risk of developing OSA than females [1, 10]. While one might expect that OSA’s association with RVO risk would also differ by sex, our stratified analysis revealed that OSA is associated with an increased risk of being diagnosed with RVO equally in both males and females. This suggests that sex does not significantly influence the association, indicating that other factors are likely driving the positive association between OSA and the risk of RVO. One such factor to consider is the presence of metabolic comorbidities, including hypertension, hypercoagulability, and atherosclerosis, which are well-established risk factors for RVO [12, 13]. While all cohorts in this study were matched for the presence of such baseline comorbidities via propensity score matching, the severity of such risk factors could not be fully controlled. Therefore, possible explanations for the observed findings are that patients in the OSA cohort had more severe metabolic conditions that naturally increased their risk of being diagnosed with RVO, or that OSA served as a synergistic risk factor that aggravated the effect of these comorbidities on the development of RVO. Replication of the findings of this study after a more granular assessment of the severity of these comorbidities, as well as additional exploration of other potential mediating factors, is necessary to confirm these hypotheses and further elucidate the mechanisms underlying the relationship between OSA and RVO. In addition, as CPAP therapy has been shown to significantly reduce the severity of metabolic conditions in patients with OSA [14–16] and prevent maculopathy among patients with diabetes [17, 18], whether early initiation of CPAP therapy can effectively mitigate the risk of RVO in patients with OSA would be a valuable area for further research.

Our race/ethnicity-stratified analysis showed that, among NHW, H/L, and Black populations, OSA is associated with a higher risk of having a new diagnosis of BRVO, but not CRVO. While both types of RVO share several risk factors [19], some studies have also highlighted nuanced differences in the degree to which each risk factors affect BRVO and CRVO. For instance, hypertension, vascular disease, and thrombotic conditions—all of which are predominant complications of OSA—have shown to have stronger association with BRVO than CRVO [20, 21]. Conversely, some studies have demonstrated that the prevalence of OSA is higher in patients with CRVO than those without [22, 23]. As the differences in the pathogenesis of BRVO and CRVO are still incompletely understood, further research is warranted.

When comparing various racial/ethnic groups, we found that having an ICD code for OSA was associated with the highest risk of receiving a new ICD code for RVO in the H/L population. One previous study comparing the overall prevalence of RVO among White, Black, Hispanic, and Chinese Americans found that race or ethnicity was not a significant risk factor for RVO development [24]. This study, however, excluded patients with cardiovascular diseases, which is one of the most significant risk factors of RVO^20^, and a pooled analysis of multiracial studies by Rogers et al. later showed that the age- and sex-standardized prevalence of RVO was indeed highest in H/L patients [25]. Currently, there is insufficient evidence to determine whether H/L ethnicity itself is an independent risk factor for RVO development or if it predisposes individuals to a greater detrimental effect of OSA on RVO risk, particularly as the higher rates of socioeconomic health hazards faced by H/L populations compared to other groups [26]. Regardless, the results of our study highlight the importance of focusing on H/L patients in future investigations of the relationship between OSA and RVO, especially given the significantly lower representation of H/L populations in existing clinical trials on RVO [27].

Conversely, there was no association between OSA and the incidence of RVO among Asian patients. This finding is notable as Asians are at higher risk for severe OSA and its complications—even at lower BMI compared to other racial groups—due to anatomical differences in craniofacial and upper airway structures [28–30]. Furthermore, while no published studies have compared Asians with other racial groups specifically on the impact of OSA and RVO, a nationwide study in Taiwan showed that the incidence of RVO was higher among OSA patients compared to controls [3, 4, 31]. The contrasting finding of this study may be explained by the heterogeneous makeup of the Asian American population, which includes not only East Asians (e.g. China, Taiwan, Hong Kong, Korea, Japan, Mongolia) but also South Asians, Southeast Asians, and others, who vary widely in their craniofacial anatomy, obesity rates, diets, and healthcare access [32–35]. A relative underdiagnosis of OSA among Asian Americans may have influenced the power of this study to detect the association between OSA and RVO, as supported by both the literature and the relatively low sample size of the Asian subgroup compared to other racial and ethnic subgroups in this study [36].

Finally, our analysis of patients with preexisting RVO suggested a positive association between OSA and the risks of having more severe RVO or complications, including ME, VH, NV, and the need for invasive treatment via PRP and intravitreal injections. Similarly, Wan et al [4]. reported that RVO patients with OSA have a higher risk of developing MO and have limited improvement in post-treatment visual acuity. Although the risk of NV was not significantly associated in their study (possibly due to limited follow-up duration) [4], the association between NV and OSA seen in our study is consistent with the well-established understanding that recurrent episodes of nocturnal hypoxia and reperfusion in OSA lead to pathological production of VEGF and oxidative stress that leads to endothelial dysfunction [4, 37, 38]. Besides, OSA has been implicated in inducing additional changes in the retinal veins, including increased tortuosity [39], decreased vessel density [40], and narrowing [41], all of which can render patients more vulnerable to clinical progression. This suggests that regular monitoring of patients with RVO with concurrent OSA and prompt treatment of OSA may play a vital role in preventing vision-threatening complications of RVO and reducing treatment burdens.

### Limitations

As a retrospective study that relied on EHR data, this study has limitations that must be acknowledged. While we strived to increase the accuracy of the OSA diagnosis by adding polysomnography testing into our inclusion criteria, detailed results of polysomnography, such as apnoea-hypopnea index (AHI) and desaturation levels, were not available. Therefore, we cannot definitively conclude that the patients in the OSA cohort were diagnosed based on objective polysomnography criteria. Similarly, the variability in OSA severity within the OSA cohort may have confounded our results. Additionally, as OSA can be significantly underdiagnosed, misclassification bias could have skewed our results to significantly underrepresent the true association between OSA and RVO.

In addition, although we included CPAP therapy as one of the covariates, we could not standardize patients’ level of adherence, duration, and responses to CPAP therapy. Consequently, variability in such factors could have additionally confounded our results. Given the relative deficiency of data on the impact of CPAP therapy on RVO, controlled trials in this area would help verify the positive associations between OSA and RVO observed in this study and previous studies and potentially offer an avenue to prevent RVO development and exacerbation.

While our large sample included patients from various racial and ethnic backgrounds, Asian patients were underrepresented. As such, it is possible that the absence of an association between OSA and RVO among Asian patients in this study could be due to underpowered analysis. Furthermore, we could not separately analyse American Indian, Alaska Native, Native Hawaiian, or other Pacific Islander populations due to sample size limitations.

Because the risk of RVO is multifactorial, unmeasured differences between the cohorts—such as in socioeconomic status, lifestyle habits, and other cardiovascular risk factors not included in the PSM—may have affected the results of our analysis. Although this study employed PSM to minimize such impact, residual effects were likely because medical record data on lifestyle habits, social determinants of health, and health-seeking behaviours are often incomplete and underreported [42, 43].

Finally, this study performed a sensitivity analysis to strengthen the validity of our analysis of patients with preexisting RVO by excluding patients with potential confounding ocular conditions. While the significantly higher proportion of events (complications or invasive treatment for RVO) in the OSA cohort supports the results of the primary analysis, the reported RRs in this sensitivity analysis may not be exact, as fewer than 10 controls had events (Table 2A) and TriNetX reports any counts of 1 to 10 as 10 to protect patient privacy.

## Conclusion

In conclusion, this analysis of large aggregated EHR data of US patients shows that OSA may be a risk factor for RVO in both males and females, and both NHW and H/L populations. This finding was particularly pronounced in H/L individuals, highlighting the need for future studies on RVO and for vision care providers to give special attention to patients with OSA in this population. While a history of OSA diagnosis was not associated with an elevated risk of a new diagnosis of RVO among Asian patients, such apparent racial/ethnic differences need further investigation.

Among patients with pre-existing RVO, OSA may be a risk factor for having more advanced RVO that may impair vision and necessitate invasive treatments. If these findings are confirmed in future prospective studies, clinicians should consider regular ophthalmology screening of all patients with OSA and, conversely, incorporating OSA screening into regular follow-up appointments for patients with RVO to allow for prompt initiation of CPAP therapy.

## Summary

### What was known before

- Emerging studies have begun to suggest that obstructive sleep apnoea (OSA)—the most common type of sleep-breathing disorder worldwide—may be a risk factor for developing retinal vein occlusion (RVO).
- However, existing evidence is largely based on small, single-institution studies and a limited number of population-wide studies that were conducted in countries with restricted diversity. As a result, whether this association holds consistently across different sexes, races, and ethnicities remains unclear.
- In addition, little is known about the role of OSA in the progression of RVO in patients already diagnosed with the condition.

### What this study adds

- This large-scale analysis of U.S. EHR data suggests that OSA may be a risk factor for RVO development in both males and females.
- Among non-Hispanic/Latino White, Hispanic/Latino, Black, and Asian patients, this association between OSA and new-onset RVO was particularly high in the Hispanic/Latino population.
- Among patients with pre-existing RVO diagnosis, those with OSA had a higher rate of being diagnosed with macular oedema, vitreous haemorrhage, and neovascularization of the eye; higher rates of undergoing panretinal photocoagulation for treatment; and received a greater number of intravitreal injections.

## Supplementary information

Supplement (eTables 1-4H)

## Data Availability

The data that support the findings of this study are available from TriNetX, LLC but restrictions apply to the availability of these data, which were used under license for the current study, and so are not publicly available. Data are however available from the authors upon reasonable request and with permission of TriNetX, LLC.

## References

[1] Young T, Peppard PE, Gottlieb DJ. Epidemiology of obstructive sleep apnea. Am J Respir Crit Care Med. 2002;165:1217–39. 11991871 10.1164/rccm.2109080

[2] Lechat B, Naik G, Reynolds A, Aishah A, Scott H, Loffler KA, et al. Multinight prevalence, variability, and diagnostic misclassification of obstructive sleep apnea. Am J Respir Crit Care Med. 2022;205:563–9. 34904935 10.1164/rccm.202107-1761OC PMC8906484

[3] Chou KT, Huang CC, Tsai DC, Chen YM, Perng DW, Shiao GM, et al. Sleep apnea and risk of retinal vein occlusion: a nationwide population-based study of Taiwanese. Am J Ophthalmol. 2012;154:200–205.e1. 22464364 10.1016/j.ajo.2012.01.011

[4] Wan W, Wu Z, Lu J, Wan W, Gao J, Su H, et al. Obstructive sleep apnea is related with the risk of retinal vein occlusion. Nat Sci Sleep. 2021;13:273–81. 33688286 10.2147/NSS.S290583 PMC7936718

[5] Glacet-Bernard A, les Jardins GL, Lasry S, Coscas G, Soubrane G, Souied E, et al. Obstructive sleep apnea among patients with retinal vein occlusion. Arch Ophthalmol. 2010;128:1533–8. 21149775 10.1001/archophthalmol.2010.272

[6] Purvin VA, Kawasaki A, Yee RD. Papilledema and obstructive sleep apnea syndrome. Arch Ophthalmol. 2000;118:1626–30. 11115256 10.1001/archopht.118.12.1626

[7] Lee AG, Golnik K, Kardon R, Wall M, Eggenberger E, Yedavally S. Sleep apnea and intracranial hypertension in men. Ophthalmology. 2002;109:482–5. 11874748 10.1016/s0161-6420(01)00987-3

[8] Hayreh SS. Anatomy and physiology of the optic nerve head. Trans Am Acad Ophthalmol Otolaryngol. 1974;78:OP240–254. 4207604

[9] Johnson DA, Ohanele C, Alcántara C, Jackson CL. The Need for Social and Environmental Determinants of Health Research to Understand and Intervene upon Racial/Ethnic Disparities in Obstructive Sleep Apnea. Clin Chest Med. 2022;43:199–216. 35659019 10.1016/j.ccm.2022.02.002 PMC9451370

[10] Lin C, Davidson T, Ancoli-Israel S. Gender differences in obstructive sleep apnea and treatment implications. Sleep Med Rev. 2008;12:481–96. 18951050 10.1016/j.smrv.2007.11.003 PMC2642982

[11] Bulloch G, Seth I, Zhu Z, Sukumar S, McNab A. Ocular manifestations of obstructive sleep apnea: a systematic review and meta-analysis. Graefes Arch Clin Exp Ophthalmol. 2024;262:19–32. 37227479 10.1007/s00417-023-06103-3 PMC10806133

[12] Song P, Xu Y, Zha M, Zhang Y, Rudan I. Global epidemiology of retinal vein occlusion: a systematic review and meta-analysis of prevalence, incidence, and risk factors. J Glob Health. 9:010427. 10.7189/jogh.09.010427 PMC6513508 31131101

[13] Klein R, Moss SE, Meuer SM, Klein BEK. The 15-year cumulative incidence of retinal vein occlusion: the Beaver Dam Eye study. Arch Ophthalmol. 2008;126:513–8. 18413521 10.1001/archopht.126.4.513

[14] Huang Z, Liu Z, Luo Q, Zhao Q, Zhao Z, Ma X, et al. Long-term effects of continuous positive airway pressure on blood pressure and prognosis in hypertensive patients with coronary heart disease and obstructive sleep apnea: a randomized controlled trial. Am J Hypertens. 2015;28:300–6. 25125635 10.1093/ajh/hpu147

[15] Cao MT, Sternbach JM, Guilleminault C. Continuous positive airway pressure therapy in obstuctive sleep apnea: benefits and alternatives. Expert Rev Respir Med. 2017;11:259–72. 28287009 10.1080/17476348.2017.1305893

[16] Faccenda JF, Mackay TW, Boon NA, Douglas NJ. Randomized placebo-controlled trial of continuous positive airway pressure on blood pressure in the sleep apnea–hypopnea syndrome. Am J Respir Crit Care Med. 2001;163:344–8. 11179104 10.1164/ajrccm.163.2.2005037

[17] West SD, Prudon B, Hughes J, Gupta R, Mohammed SB, Gerry S, et al. Continuous positive airway pressure effect on visual acuity in patients with type 2 diabetes and obstructive sleep apnoea: a multicentre randomised controlled trial. European Respiratory Journal [Internet]. 2018 Oct 1 [cited 2024 Feb 22];52. Available from: https://erj.ersjournals.com/content/52/4/1801177. 10.1183/13993003.01177-2018 PMC6203406 30166323

[18] Alali NM. The effects of obstructive sleep apnea and continuous positive airway pressure on diabetic retinopathy and maculopathy: A review and meta-analysis. 2022;45(02). Available from: https://www.teikyomedicaljournal.com/volume/TMJ/45/02/the-effects-of-obstructive-sleep-apnea-and-continuous-positive-airway-pressure-on-diabetic-retinopathy-and-maculopathy-a-review-and-meta-analysis-625109895ea7a.pdf.

[19] O’Mahoney PRA, Wong DT, Ray JG. Retinal vein occlusion and traditional risk factors for atherosclerosis. Arch Ophthalmol. 2008;126:692–9. 18474782 10.1001/archopht.126.5.692

[20] Hayreh SS, Zimmerman B, McCarthy MJ, Podhajsky P. Systemic diseases associated with various types of retinal vein occlusion. Am J Ophthalmol. 2001;131:61–77. 11162981 10.1016/s0002-9394(00)00709-1

[21] Appiah AP, Trempe CL. Differences in contributory factors among hemicentral, central, and branch retinal vein occlusions. Ophthalmology. 1989;96:364–6. 2710528 10.1016/s0161-6420(89)32884-3

[22] Wang YH, Zhang P, Chen L, Jiang Z, Li LX, He K, et al. Correlation between obstructive sleep apnea and central retinal vein occlusion. Int J Ophthalmol. 2019;12:1634–6. 31637201 10.18240/ijo.2019.10.17 PMC6796080

[23] Agard E, El Chehab H, Vie AL, Voirin N, Coste O, Dot C. Retinal vein occlusion and obstructive sleep apnea: a series of 114 patients. Acta Ophthalmol. 2018;96:e919–25. 30188014 10.1111/aos.13798

[24] Cheung N, Klein R, Wang JJ, Cotch MF, Islam AF, Klein BE, et al. Traditional and novel cardiovascular risk factors for retinal vein occlusion: the multi-ethnic study of atherosclerosis. Invest Ophthalmol Vis Sci. 2008;49:4297–302. 18539932 10.1167/iovs.08-1826 PMC2584770

[25] Rogers S, McIntosh RL, Cheung N, Lim L, Wang JJ, Mitchell P, et al. The prevalence of retinal vein occlusion: pooled data from population studies from the United States, Europe, Asia, and Australia. Ophthalmology. 2010;117:313–9.e1. 20022117 10.1016/j.ophtha.2009.07.017 PMC2945292

[26] U.S. Census Bureau. Health Insurance in the United States. [Internet]. 2023. Available from: https://data.census.gov/table?q=United+States&t=002:004:012:400:Health:Health+Insurance&g=010XX00US&y=2023.

[27] Kaakour AH, Hua HU, Rachitskaya A. Representation of race and ethnicity in randomized clinical trials of diabetic macular edema and retinal vein occlusion compared to 2010 US Census Data. JAMA Ophthalmol. 2022;140:1096–102. 36201192 10.1001/jamaophthalmol.2022.3929 PMC9539735

[28] Hnin K, Mukherjee S, Antic NA, Catcheside P, Chai-Coetzer CL, McEvoy D, et al. The impact of ethnicity on the prevalence and severity of obstructive sleep apnea. Sleep Med Rev. 2018;41:78–86. 30149931 10.1016/j.smrv.2018.01.003

[29] Li KK, Kushida C, Powell NB, Riley RW, Guilleminault C. Obstructive sleep apnea syndrome: a comparison between far-East Asian and White Men. Laryngoscope. 2000;110:1689–93. 11037826 10.1097/00005537-200010000-00022

[30] Lee RWW, Vasudavan S, Hui DS, Prvan T, Petocz P, Darendeliler MA, et al. Differences in craniofacial structures and obesity in Caucasian and Chinese Patients with Obstructive Sleep Apnea. Sleep. 2010;33:1075–80. 20815189 10.1093/sleep/33.8.1075 PMC2910536

[31] Kwon HJ, Kang EC, Lee J, Han J, Song WK. Obstructive sleep apnea in patients with branch retinal vein occlusion: a preliminary study. Korean J Ophthalmol. 2016;30:121–6. 27051260 10.3341/kjo.2016.30.2.121 PMC4820522

[32] Kwan TW, Wong SS, Hong Y, Kanaya AM, Khan SS, Hayman LL, et al. Epidemiology of diabetes and atherosclerotic cardiovascular disease among Asian American Adults: Implications, Management, and Future Directions: A Scientific Statement From the American Heart Association. Circulation. 2023;148:74–94. 37154053 10.1161/CIR.0000000000001145

[33] Bacong AM, Gibbs SL, Rosales AG, Frankland TB, Li J, Daida YG, et al. Obesity disparities among adult single-race and multiracial Asian and Pacific Islander Populations. JAMA Netw Open. 2024;7:e240734. 38502128 10.1001/jamanetworkopen.2024.0734 PMC10951735

[34] Sutherland K, Kim S, Veatch OJ, Keenan BT, Bittencourt L, Chen NH, et al. Facial and intraoral photographic traits related to sleep apnea in a clinical sample with genetic ancestry analysis. Ann ATS. 2023;20:880–90. 10.1513/AnnalsATS.202207-577OC 36780658

[35] Nguyen KH, Trivedi AN. Asian american access to care in the affordable care act era: findings from a population-based survey in California. J Gen Intern Med. 2019;34:2660–8. 31512183 10.1007/s11606-019-05328-5 PMC6848322

[36] Nandagiri V, Vannemreddy S, Spector A. Sleep disparities in Asian Americans: a comprehensive review. J Clin Sleep Med. 2023;19:393–402. 36239044 10.5664/jcsm.10330 PMC9892749

[37] Schulz R, Hummel C, Heinemann S, Seeger W, Grimminger F. Serum levels of vascular endothelial growth factor are elevated in patients with obstructive sleep apnea and severe nighttime hypoxia. Am J Respir Crit Care Med. 2002;165:67–70. 11779732 10.1164/ajrccm.165.1.2101062

[38] Jelic S, Padeletti M, Kawut SM, Higgins C, Canfield SM, Onat D, et al. Inflammation, Oxidative Stress, and Repair Capacity of the Vascular Endothelium in Obstructive Sleep Apnea. Circulation. 2008;117:2270–8. 18413499 10.1161/CIRCULATIONAHA.107.741512 PMC2844329

[39] Mohsenin A, Mohsenin V, Adelman RA. Retinal vascular tortuosity in obstructive sleep apnea. Clin Ophthalmol. 2013;7:787–92. 10.2147/OPTH.S41795. 10.2147/OPTH.S41795 PMC3639720 23641149

[40] Yu J, Xiao K, Huang J, Sun X, Jiang C. Reduced retinal vessel density in obstructive sleep apnea syndrome patients: an optical coherence tomography angiography study. Investig Ophthalmol Vis Sci. 2017;58:3506–12. 28715584 10.1167/iovs.17-21414

[41] Tong JY, Golzan M, Georgevsky D, Williamson JP, Graham SL, Farah CS, et al. Quantitative retinal vascular changes in obstructive sleep apnea. Am J Ophthalmol. 2017;182:72–80. 28734812 10.1016/j.ajo.2017.07.012

[42] Guo Y, Chen Z, Xu K, George TJ, Wu Y, Hogan W, et al. International classification of diseases, tenth revision, clinical modification social determinants of health codes are poorly used in electronic health records. Med (Balt). 2020;99:e23818. 10.1097/MD.0000000000023818 PMC7769291 33350768

[43] Truong HP, Luke AA, Hammond G, Wadhera RK, Reidhead M, Maddox KEJ. Utilization of social determinants of health ICD-10 Z-codes among hospitalized patients in the United States, 2016–2017. Med Care. 2020;58:1037–43. 32925453 10.1097/MLR.0000000000001418 PMC7666017

