# Supplementary material for: Associations between obstructive sleep apnoea and the development and severity of retinal vein occlusion
Source: Eye (Lond). 2025 Jul 16;39(13):2584–90. doi: 10.1038/s41433-025-03900-4 (PMC12402211; doi:10.1038/s41433-025-03900-4)
Supplement: Supplementary file 1 — Supplement (eTables 1-4H) [file 41433_2025_3900_MOESM1_ESM.docx]

Supplemental Online Content

Hejin Jeong, Jacqueline K. Shaia, David C. Kaelber, Katherine E. Talcott, Rishi P. Singh

Associations between Obstructive Sleep Apnea and the Development and Severity of Retinal Vein Occlusion

eTable 1: Relevant codes used for the cohort construction and for outcome measures

eTable 2: Covariates and their relevant codes for propensity score matching

eTable 3: Summary of the inclusion and exclusion criteria for the analysis of the risk of RVO development among non-Hispanic White patients

eTable 4A: Results of the propensity score matching analyses performed on OSA-naïve females

eTable 4B: Results of the propensity score matching analyses performed on OSA-naïve males

eTable 4C: Results of the propensity score matching analyses performed on OSA-naïve non-Hispanic or non-Latino White patients

eTable 4D: Results of the propensity score matching analyses performed on OSA-naïve Hispanic/Latino patients

eTable 4E: Results of the propensity score matching analyses performed on OSA-naïve Black patients

eTable 4F: Results of the propensity score matching analyses performed on OSA-naïve Asian patients

eTable 4G: Results of the propensity score matching analyses performed on patients with a previous history of any retinal vein occlusion

eTable 4H: Results of the propensity score matching analyses performed on patients with a previous history of any retinal vein occlusion but without confounding ocular disorders (sensitivity analysis)

eTable 1: Relevant codes used for the cohort construction and for outcome measures

| **Name** | **Code System** | **Relevant code** |
| --- | --- | --- |
| Age ≥18 years |  |  |
| *Sex* |  |  |
| Male |  |  |
| Female |  |  |
| *Race and Ethnicity* |  |  |
| Non-Hispanic and non-Latino White |  |  |
| Hispanic or Latino |  |  |
| Black |  |  |
| Asian |  |  |
| Vision |  |  |
| Vision care |  |  |
| Encounter for examination of eyes and vision | ICD-10 | Z01.0 |
| Encounter for screening for eye and ear disorders | ICD-10 | Z13.5 |
| Ophthalmology Services and Procedures | CPT | 1012793 |
| Obstructive sleep apnea | ICD-10 | G47.33 |
| Sleep apnea | ICD-10 | G47.3 |
| Sleep disorder, unspecified | ICD-10 | G47.9 |
| Polysomnography | CPT | 1013314 |
| *Outcome measures for the development of retinal vein occlusion* | | |
| Any retinal vein occlusion | ICD-10 | H34.81, H34.83 |
| Central retinal vein occlusion | ICD-10 | H34.81 |
| Branch retinal vein occlusion | ICD-10 | H34.83 |
| *Outcome measures for the progression of retinal vein occlusion* | | |
| Macular edema | ICD-10 | H35.81, H35.35, H34.8110, H34.8120, H34.8130, H34.8190, H34.8310, H34.8320, H34.8330, H34.8390 |
| Vitreous hemorrhage | ICD-10 | H43.1 |
| Neovascularization of the eye | ICD-10 | H35.05, H21.1, H40.89, H34.8111, H34.8121, H34.8131, H34.8191, H34.8311, H34.8321, H34.8331, H34.8391 |
| Pars plana vitrectomy | CPT | 1014238 |
| Panretinal Photocoagulation | CPT | 67228 |
| *Number of Intravitreal injections* |  |  |
| Ophthalmic anti-VEGF agents | RxNorm | 1232150, 2204915, 253337, 2591519, 498509, 595060 |
| Ophthalmic corticosteroids | RxNorm | 10759, 1514, 19831, 22396, 2878, 3264, 5492, 6902, 7910, 8638, 8640 |
| Intravitreal injection of a pharmacologic agent | CPT | 67028 |
| *Exclusions for the supplementary analysis on the progression of retinal vein occlusion* | | |
| Severe non-proliferative diabetic retinopathy or proliferative diabetic retinopathy | ICD-10 | E10.34, E10.35, E11.34, E11.35 |
| Retinoschisis and retinal cysts | ICD-10 | H33.1 |
| Exudative age-related macular degeneration | ICD-10 | H35.32 |
| Macular cyst, hole, or pseudohole | ICD-10 | H35.34 |
| Puckering of macula | ICD-10 | H35.37 |
| Central serous retinopathy | ICD-10 | H35.71 |
| Glaucoma | ICD-10 | H40-H42 |
| Purulent endophthalmitis | ICD-10 | H44.0 |
| Other endophthalmitis | ICD-10 | H44.1 |
| Degenerative myopia | ICD-10 | H44.2 |
| Intraoperative and postprocedural complications and disorders of eye and adnexa, not elsewhere classified | ICD-10 | H59 |
| Injury of eye and orbit | ICD-10 | S05 |
| Intraocular Lens Procedures | CPT | 1018495 |

ICD-10: International Classification of Diseases, 10th Revision; LOINC: Logical Observation Identifier Names and Codes; CPT: Current Procedural Terminology

eTable 2: Covariates and their relevant codes for propensity score matching

| **Covariate** | **Code System** | **Relevant code** |
| --- | --- | --- |
| Age at Index |  |  |
| Female |  |  |
| Hispanic or Latino |  |  |
| *Race* |  |  |
| White |  |  |
| Black or African American |  |  |
| Asian |  |  |
| Native Hawaiian or Other Pacific Islander |  |  |
| American Indian or Alaska Native |  |  |
| Other known race (unspecified) |  |  |
| Body Mass Index (kg/m^2^) | LOINC | 39156-5 |
| <18.5 |  |  |
| 18.5-24.9 |  |  |
| 25-29.9 |  |  |
| 30-34.9 |  |  |
| 35-40 |  |  |
| >40.0 |  |  |
| Hemoglobin A1c (%) | LOINC | 4548-4 |
| <7 |  |  |
| 7-10 |  |  |
| ≥10 |  |  |
| Persons with potential health hazards related to family and personal history and certain conditions influencing health status | ICD-10 | Z55-Z65 |
| Tobacco use | ICD-10 | Z72.0 |
| Alcohol related disorders | ICD-10 | F10 |
| Diseases of the circulatory system | ICD-10 | I00-I99 |
| Hypertensive diseases | ICD-10 | I10-I1A |
| Ischemic heart diseases | ICD-10 | I20-I25 |
| Diseases of arteries, arterioles and capillaries | ICD-10 | I70-I79 |
| Disorders of lipoprotein metabolism and other lipidemias | ICD-10 | E78 |
| Chronic lower respiratory diseases | ICD-10 | J40-J4A |
| Pulmonary heart disease and diseases of pulmonary circulation | ICD-10 | I26-I28 |
| Diabetes mellitus | ICD-10 | E08-E13 |
| Cerebrovascular diseases | ICD-10 | I60-I69 |
| Acute kidney failure and chronic kidney disease | ICD-10 | N17-N19 |
| Primary thrombophilia | ICD-10 | D68.5 |
| Other thrombophilia | ICD-10 | D68.6 |
| Polycythemia vera | ICD-10 | D45 |
| Homocystinuria | ICD-10 | E72.11 |
| Malignant immunoproliferative diseases and certain other B-cell lymphomas | ICD-10 | C88 |
| Multiple myeloma and malignant plasma cell neoplasms | ICD-10 | C90 |
| Insomnia | ICD-10 | G47.0 |
| Circadian rhythm sleep disorders | ICD-10 | G47.2 |
| Sleep disorders not due to a substance or known physiological condition | ICD-10 | F51 |
| Glaucoma | ICD-10 | H40-H42 |
| Type 1 diabetes mellitus with ophthalmic complications | ICD-10 | E10.3 |
| Type 2 diabetes mellitus with ophthalmic complications | ICD-10 | E11.3 |
| Nonexudative age-related macular degeneration | ICD-10 | H35.31 |
| Exudative age-related macular degeneration | ICD-10 | H35.32 |
| Continuous positive airway pressure^a^ | CPT | 94660 |
| *Medications* |  |  |
| Antithrombotic agents | ATC | B01 |
| Antihypertensives | ATC | C02 |
| Lipid modifying agents | ATC | C10 |
| Hypnotics and sedatives | ATC | N05C |
| Estrogens | ATC | G03C |
| Central retinal vein occlusion^a^ | ICD-10 | H34.81 |
| Branch retinal vein occlusion^a^ | ICD-10 | H34.83 |

^a^Only included in the analysis on patients with a preeixsitng history of retinal vein occlusion

ICD-10: International Classification of Diseases, 10th Revision; LOINC: Logical Observation Identifiers Names and Codes; CPT: Current Procedural Terminology; ATC: Anatomical Therapeutic Chemical

eTable 3: Summary of the inclusion and exclusion criteria for the analysis of the risk of RVO development among non-Hispanic White patients

| **OSA cohort** | **Control cohort** |
| --- | --- |
| ***Inclusion Criteria*** |  |
| Age≥18 years | Age≥18 years |
| *Race:* White | *Race:* White |
| Ethnicity: Not Hispanic or Latino | Ethnicity: Not Hispanic or Latino |
| Polysomnography |  |
| History of OSA |  |
| ***Exclusion Criteria*** |  |
| History of CRVO or BRVO before the index date | History of CRVO or BRVO before the index date |
| *Race / Ethnicity* | *Race / Ethnicity* |
| Black | Black |
| Asian | Asian |
| American Indian or Alaska Native | American Indian or Alaska Native |
| Native Hawaiian or Other Pacific Islander | Native Hawaiian or Other Pacific Islander |
| Unspecified race | Unspecified race |
| Hispanic or Latino | Hispanic or Latino |
|  | History of sleep apnea |
|  | History of unspecified sleep disorder |

OSA = obstructive sleep apnea; RVO = retinal vein occlusion; CRVO = central retinal vein occlusion; BRVO = branch retinal vein occlusion

eTable 4A: Results of the propensity score matching analyses performed on OSA-naïve females

|  | **Before Matching** | | | | | **After Matching** | | | | |
| --- | --- | --- | --- | --- | --- | --- | --- | --- | --- | --- |
|  | **Obstructive Sleep Apnea** | | **Control** | | **SMD** | **Obstructive Sleep Apnea** | | **Control** | | **SMD** |
| Number of Patients | 82,670 | | 1,724,087 | |  | 74,018 | | 74,018 | |  |
| Age at Index | 55.39 | (15.87) | 21.24 | (21.24) | 0.276 | 55.21 | (16.08) | 55.99 | (17.38) | 0.046 |
| *Sex* |  |  |  |  |  |  |  |  |  |  |
| Male | 0 | (0.00%) | 0 | (0.00%) |  | 0 | (0.00%) | 0 | (0.00%) |  |
| Female | 82,670 | (100.00%) | 1,724,087 | (100.00%) |  | 74,018 | (100.00%) | 74,018 | (100.00%) |  |
| *Ethnicity* |  |  |  |  |  |  |  |  |  |  |
| Hispanic or Latino | 9,138 | (11.05%) | 184,685 | (10.71%) | 0.011 | 8,477 | (11.45%) | 8,763 | (11.84%) | 0.012 |
| Not Hispanic or Latino | 65,686 | (79.46%) | 1,260,314 | (73.10%) | 0.150 | 58,464 | (78.99%) | 56,749 | (76.67%) | 0.056 |
| *Race* |  |  |  |  |  |  |  |  |  |  |
| White | 46,360 | (56.08%) | 1,037,032 | (60.15%) | 0.083 | 41,973 | (56.71%) | 43,037 | (58.14%) | 0.029 |
| Black or African American | 22,346 | (27.03%) | 252,093 | (14.62%) | 0.309 | 19,241 | (26.00%) | 18,816 | (25.42%) | 0.013 |
| Asian | 1,505 | (1.82%) | 81,671 | (4.74%) | 0.164 | 1,475 | (1.99%) | 1,397 | (1.89%) | 0.008 |
| Native Hawaiian or Other Pacific Islander | 176 | (0.21%) | 4,632 | (0.27%) | 0.011 | 158 | (0.21%) | 179 | (0.24%) | 0.006 |
| American Indian or Alaska Native | 414 | (0.50%) | 6,644 | (0.39%) | 0.017 | 367 | (0.50%) | 349 | (0.47%) | 0.004 |
| Other known race (unspecified) | 2,627 | (3.18%) | 90,804 | (5.27%) | 0.104 | 2,426 | (3.28%) | 2,343 | (3.17%) | 0.006 |
| *Body Mass Index (kg/m^2^)* | 64,988 | (78.61%) | 816,788 | (47.38%) | 1.029 | 56,969 | (76.97%) | 57,671 | (77.91%) | 0.297 |
| <18.5 | 4,208 | (5.09%) | 83,616 | (4.85%) | 0.011 | 3,649 | (4.93%) | 3,529 | (4.77%) | 0.008 |
| 18.5-24.9 | 15,259 | (18.46%) | 387,851 | (22.50%) | 0.100 | 14,071 | (19.01%) | 14,938 | (20.18%) | 0.030 |
| 25-29.9 | 26,405 | (31.94%) | 357,687 | (20.75%) | 0.256 | 23,790 | (32.14%) | 25,435 | (34.36%) | 0.047 |
| 30-34.9 | 33,077 | (40.01%) | 241,153 | (13.99%) | 0.613 | 28,508 | (38.51%) | 29,763 | (40.21%) | 0.035 |
| 35-40 | 30,933 | (37.42%) | 129,609 | (7.52%) | 0.767 | 25,296 | (34.18%) | 25,338 | (34.23%) | 0.001 |
| >40.0 | 29,925 | (36.20%) | 81,437 | (4.72%) | 0.847 | 23,598 | (31.88%) | 22,834 | (30.85%) | 0.022 |
| *Hemoglobin A1c (%)* | 49,831 | (60.28%) | 327,057 | (18.97%) | 0.038 | 42,530 | (57.46%) | 43,419 | (58.66%) | 0.010 |
| <7 | 6,634 | (8.02%) | 35,486 | (2.06%) | 0.275 | 5,445 | (7.36%) | 5,300 | (7.16%) | 0.008 |
| 7-10 | 16,143 | (19.53%) | 81,128 | (4.71%) | 0.466 | 13,241 | (17.89%) | 13,277 | (17.94%) | 0.001 |
| ≥10 | 45,222 | (54.70%) | 284,164 | (16.48%) | 0.871 | 38,390 | (51.87%) | 39,621 | (53.53%) | 0.033 |
| Persons with potential health hazards related to family and personal history and certain conditions influencing health status | 8,433 | (10.20%) | 42,425 | (2.46%) | 0.322 | 6,590 | (8.90%) | 6,447 | (8.71%) | 0.007 |
| Tobacco use | 4,920 | (5.95%) | 21,979 | (1.27%) | 0.253 | 3,916 | (5.29%) | 3,866 | (5.22%) | 0.003 |
| Alcohol related disorders | 4,126 | (4.99%) | 26,556 | (1.54%) | 0.195 | 3,393 | (4.58%) | 3,322 | (4.49%) | 0.005 |
| Diseases of the circulatory system | 69,268 | (83.79%) | 597,896 | (34.68%) | 1.154 | 60,811 | (82.16%) | 62,771 | (84.81%) | 0.071 |
| Hypertensive diseases | 58,071 | (70.24%) | 441,782 | (25.62%) | 0.998 | 50,348 | (68.02%) | 51,542 | (69.63%) | 0.035 |
| Ischemic heart diseases | 19,032 | (23.02%) | 84,037 | (4.87%) | 0.543 | 15,235 | (20.58%) | 15,090 | (20.39%) | 0.005 |
| Diseases of arteries, arterioles and capillaries | 17,730 | (21.45%) | 100,734 | (5.84%) | 0.467 | 14,633 | (19.77%) | 14,650 | (19.79%) | 0.001 |
| Disorders of lipoprotein metabolism and other lipidemias | 54,158 | (65.51%) | 398,194 | (23.10%) | 0.944 | 46,806 | (63.24%) | 48,039 | (64.90%) | 0.035 |
| Chronic lower respiratory diseases | 40,475 | (48.96%) | 225,764 | (13.09%) | 0.841 | 33,635 | (45.44%) | 33,806 | (45.67%) | 0.005 |
| Pulmonary heart disease and diseases of pulmonary circulation | 10,639 | (12.87%) | 26,874 | (1.56%) | 0.448 | 7,692 | (10.39%) | 7,198 | (9.72%) | 0.022 |
| Diabetes mellitus | 35,257 | (42.65%) | 280,025 | (16.24%) | 0.605 | 29,768 | (40.22%) | 29,770 | (40.22%) | 0.000 |
| Cerebrovascular diseases | 13,143 | (15.90%) | 79,150 | (4.59%) | 0.380 | 10,871 | (14.69%) | 10,898 | (14.72%) | 0.001 |
| Acute kidney failure and chronic kidney disease | 15,562 | (18.82%) | 74,990 | (4.35%) | 0.464 | 12,323 | (16.65%) | 12,263 | (16.57%) | 0.002 |
| Primary thrombophilia | 1,462 | (1.77%) | 7,276 | (0.42%) | 0.130 | 1,133 | (1.53%) | 1,109 | (1.50%) | 0.003 |
| Other thrombophilia | 1,277 | (1.54%) | 6,612 | (0.38%) | 0.119 | 1,003 | (1.36%) | 993 | (1.34%) | 0.001 |
| Polycythemia vera | 366 | (0.44%) | 1,241 | (0.07%) | 0.073 | 298 | (0.40%) | 197 | (0.27%) | 0.024 |
| Homocystinuria | 268 | (0.32%) | 1,274 | (0.07%) | 0.056 | 217 | (0.29%) | 181 | (0.24%) | 0.009 |
| Malignant immunoproliferative diseases and certain other B-cell lymphomas | 72 | (0.09%) | 760 | (0.04%) | 0.017 | 64 | (0.09%) | 80 | (0.11%) | 0.007 |
| Multiple myeloma and malignant plasma cell neoplasms | 296 | (0.36%) | 2,904 | (0.17%) | 0.037 | 252 | (0.34%) | 279 | (0.38%) | 0.006 |
| Insomnia | 26,042 | (31.50%) | 79,514 | (4.61%) | 0.746 | 20,381 | (27.54%) | 20,393 | (27.55%) | 0.000 |
| Circadian rhythm sleep disorders | 2,948 | (3.57%) | 1,915 | (0.11%) | 0.259 | 1,414 | (1.91%) | 1,168 | (1.58%) | 0.025 |
| Sleep disorders not due to a substance or known physiological condition | 10,539 | (12.75%) | 21,579 | (1.25%) | 0.462 | 7,496 | (10.13%) | 7,307 | (9.87%) | 0.009 |
| Glaucoma | 16,399 | (19.84%) | 241,099 | (13.98%) | 0.157 | 14,240 | (19.24%) | 13,727 | (18.55%) | 0.018 |
| Type 1 diabetes mellitus with ophthalmic complications | 961 | (1.16%) | 7,864 | (0.46%) | 0.079 | 781 | (1.06%) | 768 | (1.04%) | 0.002 |
| Type 2 diabetes mellitus with ophthalmic complications | 8,132 | (9.84%) | 65,708 | (3.81%) | 0.241 | 6,717 | (9.07%) | 6,399 | (8.65%) | 0.015 |
| Nonexudative age-related macular degeneration | 1,877 | (2.27%) | 39,724 | (2.30%) | 0.002 | 1,704 | (2.30%) | 1,715 | (2.32%) | 0.001 |
| Exudative age-related macular degeneration | 562 | (0.68%) | 17,437 | (1.01%) | 0.036 | 500 | (0.68%) | 528 | (0.71%) | 0.005 |
| Continuous positive airway pressure ventilation therapy | 5,115 | (6.19%) | 2,144 | (0.12%) | 0.352 | 2,073 | (2.80%) | 1,595 | (2.15%) | 0.042 |
| *Medications* |  |  |  |  |  |  |  |  |  |  |
| Antithrombotic agents | 46,827 | (56.64%) | 330,081 | (19.15%) | 0.838 | 39,760 | (53.72%) | 40,635 | (54.90%) | 0.024 |
| Antihypertensives | 15,596 | (18.87%) | 74,243 | (4.31%) | 0.467 | 12,306 | (16.63%) | 12,097 | (16.34%) | 0.008 |
| Lipid modifying agents | 41,801 | (50.56%) | 317,115 | (18.39%) | 0.719 | 35,768 | (48.32%) | 36,528 | (49.35%) | 0.021 |
| Hypnotics and sedatives | 49,276 | (59.61%) | 289,319 | (16.78%) | 0.982 | 41,503 | (56.07%) | 42,303 | (57.15%) | 0.022 |
| Estrogens | 22,036 | (26.66%) | 215,794 | (12.52%) | 0.362 | 18,972 | (25.63%) | 19,287 | (26.06%) | 0.010 |

SMD = standardized mean difference

eTable 4B: Results of the propensity score matching analyses performed on OSA-naïve males

|  | **Before Matching** | | | | | **After Matching** | | | | |
| --- | --- | --- | --- | --- | --- | --- | --- | --- | --- | --- |
|  | **Obstructive Sleep Apnea** | | **Control** | | **SMD** | **Obstructive Sleep Apnea** | | **Control** | | **SMD** |
| Number of Patients | 74,137 | | 1,309,112 | |  | 67,174 | | 67,174 | |  |
| Age at Index | 55.98 | (16.78) | 21.68 | (21.68) | 0.356 | 55.70 | (17.02) | 56.76 | (17.40) | 0.061 |
| *Sex* |  |  |  |  |  |  |  |  |  |  |
| Male | 74,137 | (100.00%) | 1,309,112 | (100.00%) |  | 67,174 | (100.00%) | 67,174 | (100.00%) |  |
| Female | 0 | (0.00%) | 0 | (0.00%) |  | 0 | (0.00%) | 0 | (0.00%) |  |
| *Ethnicity* |  |  |  |  |  |  |  |  |  |  |
| Hispanic or Latino | 7,591 | (10.24%) | 141,443 | (10.80%) | 0.018 | 7,099 | (10.57%) | 7,228 | (10.76%) | 0.006 |
| Not Hispanic or Latino | 60,286 | (81.32%) | 953,600 | (72.84%) | 0.203 | 54,186 | (80.67%) | 54,320 | (80.86%) | 0.005 |
| *Race* |  |  |  |  |  |  |  |  |  |  |
| White | 49,491 | (66.76%) | 795,291 | (60.75%) | 0.125 | 44,651 | (66.47%) | 45,311 | (67.45%) | 0.021 |
| Black or African American | 12,324 | (16.62%) | 178,889 | (13.66%) | 0.083 | 10,992 | (16.36%) | 10,859 | (16.17%) | 0.005 |
| Asian | 2,048 | (2.76%) | 56,862 | (4.34%) | 0.085 | 1,983 | (2.95%) | 1,979 | (2.95%) | 0.000 |
| Native Hawaiian or Other Pacific Islander | 198 | (0.27%) | 3,331 | (0.25%) | 0.002 | 182 | (0.27%) | 160 | (0.24%) | 0.006 |
| American Indian or Alaska Native | 276 | (0.37%) | 4,641 | (0.35%) | 0.003 | 245 | (0.36%) | 246 | (0.37%) | 0.000 |
| Other known race (unspecified) | 2,548 | (3.44%) | 71,820 | (5.49%) | 0.099 | 2,381 | (3.54%) | 2,243 | (3.34%) | 0.011 |
| *Body Mass Index (kg/m^2^)* | 58,037 | (78.28%) | 600,744 | (45.89%) | 0.923 | 51,418 | (76.54%) | 52,357 | (77.94%) | 0.270 |
| <18.5 | 3,854 | (5.20%) | 63,071 | (4.82%) | 0.017 | 3,301 | (4.91%) | 3,116 | (4.64%) | 0.013 |
| 18.5-24.9 | 13,323 | (17.97%) | 249,769 | (19.08%) | 0.029 | 12,322 | (18.34%) | 12,993 | (19.34%) | 0.026 |
| 25-29.9 | 28,966 | (39.07%) | 310,069 | (23.69%) | 0.336 | 26,431 | (39.35%) | 28,816 | (42.90%) | 0.072 |
| 30-34.9 | 32,267 | (43.52%) | 176,142 | (13.46%) | 0.707 | 27,807 | (41.40%) | 29,462 | (43.86%) | 0.050 |
| 35-40 | 23,295 | (31.42%) | 66,192 | (5.06%) | 0.726 | 18,520 | (27.57%) | 18,282 | (27.22%) | 0.008 |
| >40.0 | 17,063 | (23.02%) | 30,384 | (2.32%) | 0.655 | 12,663 | (18.85%) | 11,767 | (17.52%) | 0.035 |
| *Hemoglobin A1c (%)* | 42,114 | (56.81%) | 245,519 | (18.75%) | 0.052 | 36,353 | (54.12%) | 37,284 | (55.50%) | 0.011 |
| <7 | 6,885 | (9.29%) | 37,852 | (2.89%) | 0.270 | 5,795 | (8.63%) | 5,765 | (8.58%) | 0.002 |
| 7-10 | 16,013 | (21.60%) | 76,902 | (5.87%) | 0.469 | 13,389 | (19.93%) | 13,455 | (20.03%) | 0.002 |
| ≥10 | 37,570 | (50.68%) | 202,364 | (15.46%) | 0.807 | 32,208 | (47.95%) | 33,069 | (49.23%) | 0.026 |
| Persons with potential health hazards related to family and personal history and certain conditions influencing health status | 4,648 | (6.27%) | 25,027 | (1.91%) | 0.221 | 3,743 | (5.57%) | 3,682 | (5.48%) | 0.004 |
| Tobacco use | 4,145 | (5.59%) | 23,548 | (1.80%) | 0.202 | 3,469 | (5.16%) | 3,470 | (5.17%) | 0.000 |
| Alcohol related disorders | 7,380 | (9.95%) | 48,978 | (3.74%) | 0.248 | 6,253 | (9.31%) | 6,235 | (9.28%) | 0.001 |
| Diseases of the circulatory system | 61,405 | (82.83%) | 452,329 | (34.55%) | 1.125 | 54,581 | (81.25%) | 56,768 | (84.51%) | 0.087 |
| Hypertensive diseases | 53,089 | (71.61%) | 349,516 | (26.70%) | 1.005 | 46,684 | (69.50%) | 48,280 | (71.87%) | 0.052 |
| Ischemic heart diseases | 23,204 | (31.30%) | 105,289 | (8.04%) | 0.612 | 19,397 | (28.88%) | 19,544 | (29.09%) | 0.005 |
| Diseases of arteries, arterioles and capillaries | 17,407 | (23.48%) | 77,405 | (5.91%) | 0.512 | 14,484 | (21.56%) | 14,566 | (21.68%) | 0.003 |
| Disorders of lipoprotein metabolism and other lipidemias | 52,668 | (71.04%) | 319,526 | (24.41%) | 1.056 | 46,331 | (68.97%) | 48,127 | (71.65%) | 0.059 |
| Chronic lower respiratory diseases | 27,526 | (37.13%) | 139,017 | (10.62%) | 0.654 | 22,773 | (33.90%) | 22,656 | (33.73%) | 0.004 |
| Pulmonary heart disease and diseases of pulmonary circulation | 8,271 | (11.16%) | 19,382 | (1.48%) | 0.406 | 6,051 | (9.01%) | 5,732 | (8.53%) | 0.017 |
| Diabetes mellitus | 31,772 | (42.86%) | 250,848 | (19.16%) | 0.530 | 27,365 | (40.74%) | 27,624 | (41.12%) | 0.008 |
| Cerebrovascular diseases | 12,479 | (16.83%) | 66,638 | (5.09%) | 0.383 | 10,606 | (15.79%) | 10,762 | (16.02%) | 0.006 |
| Acute kidney failure and chronic kidney disease | 17,268 | (23.29%) | 80,052 | (6.11%) | 0.500 | 14,120 | (21.02%) | 14,112 | (21.01%) | 0.000 |
| Primary thrombophilia | 960 | (1.29%) | 3,540 | (0.27%) | 0.116 | 776 | (1.16%) | 750 | (1.12%) | 0.004 |
| Other thrombophilia | 842 | (1.14%) | 2,990 | (0.23%) | 0.110 | 685 | (1.02%) | 661 | (0.98%) | 0.004 |
| Polycythemia vera | 689 | (0.93%) | 1,480 | (0.11%) | 0.114 | 498 | (0.74%) | 477 | (0.71%) | 0.004 |
| Homocystinuria | 208 | (0.28%) | 739 | (0.06%) | 0.055 | 165 | (0.25%) | 159 | (0.24%) | 0.002 |
| Malignant immunoproliferative diseases and certain other B-cell lymphomas | 90 | (0.12%) | 689 | (0.05%) | 0.023 | 77 | (0.11%) | 71 | (0.11%) | 0.003 |
| Multiple myeloma and malignant plasma cell neoplasms | 411 | (0.55%) | 2,907 | (0.22%) | 0.053 | 350 | (0.52%) | 350 | (0.52%) | 0.000 |
| Insomnia | 16,789 | (22.65%) | 39,304 | (3.00%) | 0.615 | 13,043 | (19.42%) | 12,789 | (19.04%) | 0.010 |
| Circadian rhythm sleep disorders | 2,274 | (3.07%) | 1,262 | (0.10%) | 0.240 | 1,106 | (1.65%) | 861 | (1.28%) | 0.030 |
| Sleep disorders not due to a substance or known physiological condition | 6,791 | (9.16%) | 10,353 | (0.79%) | 0.392 | 4,832 | (7.19%) | 4,477 | (6.66%) | 0.021 |
| Glaucoma | 13,912 | (18.77%) | 183,224 | (14.00%) | 0.129 | 12,390 | (18.44%) | 12,197 | (18.16%) | 0.007 |
| Type 1 diabetes mellitus with ophthalmic complications | 926 | (1.25%) | 7,711 | (0.59%) | 0.069 | 774 | (1.15%) | 727 | (1.08%) | 0.007 |
| Type 2 diabetes mellitus with ophthalmic complications | 7,588 | (10.24%) | 66,323 | (5.07%) | 0.195 | 6,441 | (9.59%) | 6,138 | (9.14%) | 0.015 |
| Nonexudative age-related macular degeneration | 1,845 | (2.49%) | 23,972 | (1.83%) | 0.045 | 1,634 | (2.43%) | 1,650 | (2.46%) | 0.002 |
| Exudative age-related macular degeneration | 540 | (0.73%) | 10,582 | (0.81%) | 0.009 | 487 | (0.72%) | 462 | (0.69%) | 0.004 |
| Continuous positive airway pressure ventilation therapy | 5,406 | (7.29%) | 2,496 | (0.19%) | 0.381 | 2,474 | (3.68%) | 1,836 | (2.73%) | 0.054 |
| *Medications* |  |  |  |  |  |  |  |  |  |  |
| Antithrombotic agents | 43,262 | (58.35%) | 276,578 | (21.13%) | 0.823 | 37,390 | (55.66%) | 38,518 | (57.34%) | 0.034 |
| Antihypertensives | 14,527 | (19.59%) | 68,857 | (5.26%) | 0.445 | 11,764 | (17.51%) | 11,694 | (17.41%) | 0.003 |
| Lipid modifying agents | 42,796 | (57.73%) | 284,054 | (21.70%) | 0.792 | 37,407 | (55.69%) | 38,527 | (57.35%) | 0.034 |
| Hypnotics and sedatives | 38,978 | (52.58%) | 199,317 | (15.23%) | 0.859 | 33,036 | (49.18%) | 33,634 | (50.07%) | 0.018 |
| Estrogens | 230 | (0.31%) | 1,200 | (0.09%) | 0.049 | 184 | (0.27%) | 189 | (0.28%) | 0.001 |

SMD = standardized mean difference

eTable 4C: Results of the propensity score matching analyses performed on OSA-naïve non-Hispanic or non-Latino White patients

|  | **Before Matching** | | | | | **After Matching** | | | | |
| --- | --- | --- | --- | --- | --- | --- | --- | --- | --- | --- |
|  | **Obstructive Sleep Apnea** | | **Control** | | **SMD** | **Obstructive Sleep Apnea** | | **Control** | | **SMD** |
| Number of Patients | 80,913 | | 1,460,706 | |  | 73,062 | | 73,062 | |  |
| Age at Index | 58.12 | (15.82) | 21.60 | (21.60) | 0.307 | 57.95 | (16.01) | 58.93 | (16.94) | 0.060 |
| *Sex* |  |  |  |  |  |  |  |  |  |  |
| Male | 41,716 | (51.56%) | 616,379 | (42.20%) | 0.188 | 37,486 | (51.31%) | 37,250 | (50.98%) | 0.006 |
| Female | 39,183 | (48.43%) | 844,027 | (57.78%) | 0.188 | 35,564 | (48.68%) | 35,795 | (48.99%) | 0.006 |
| *Ethnicity* |  |  |  |  |  |  |  |  |  |  |
| Hispanic or Latino | 0 | (0.00%) | 0 | (0.00%) |  | 0 | (0.00%) | 0 | (0.00%) |  |
| Not Hispanic or Latino | 80,913 | (100.00%) | 1,460,706 | (100.00%) |  | 73,062 | (100.00%) | 73,062 | (100.00%) |  |
| *Race* |  |  |  |  |  |  |  |  |  |  |
| White | 80,913 | (100.00%) | 1,460,706 | (100.00%) |  | 73,062 | (100.00%) | 73,062 | (100.00%) |  |
| Black or African American | 0 | (0.00%) | 0 | (0.00%) |  | 0 | (0.00%) | 0 | (0.00%) |  |
| Asian | 0 | (0.00%) | 0 | (0.00%) |  | 0 | (0.00%) | 0 | (0.00%) |  |
| Native Hawaiian or Other Pacific Islander | 0 | (0.00%) | 0 | (0.00%) |  | 0 | (0.00%) | 0 | (0.00%) |  |
| American Indian or Alaska Native | 0 | (0.00%) | 0 | (0.00%) |  | 0 | (0.00%) | 0 | (0.00%) |  |
| Other known race (unspecified) | 0 | (0.00%) | 0 | (0.00%) |  | 0 | (0.00%) | 0 | (0.00%) |  |
| *Body Mass Index (kg/m^2^)* | 68,895 | (85.15%) | 757,377 | (51.85%) | 0.940 | 61,343 | (83.96%) | 62,010 | (84.87%) | 0.268 |
| <18.5 | 3,816 | (4.72%) | 76,512 | (5.24%) | 0.024 | 3,386 | (4.63%) | 3,173 | (4.34%) | 0.014 |
| 18.5-24.9 | 16,681 | (20.62%) | 353,865 | (24.23%) | 0.087 | 15,522 | (21.24%) | 16,536 | (22.63%) | 0.034 |
| 25-29.9 | 33,133 | (40.95%) | 364,011 | (24.92%) | 0.346 | 30,216 | (41.36%) | 32,680 | (44.73%) | 0.068 |
| 30-34.9 | 37,834 | (46.76%) | 219,623 | (15.04%) | 0.731 | 32,840 | (44.95%) | 34,513 | (47.24%) | 0.046 |
| 35-40 | 29,925 | (36.98%) | 100,371 | (6.87%) | 0.781 | 24,406 | (33.40%) | 24,353 | (33.33%) | 0.002 |
| >40.0 | 24,002 | (29.66%) | 55,240 | (3.78%) | 0.739 | 18,666 | (25.55%) | 17,992 | (24.63%) | 0.021 |
| *Hemoglobin A1c (%)* | 45,498 | (56.23%) | 247,620 | (16.95%) | 0.045 | 39,071 | (53.48%) | 39,842 | (54.53%) | 0.013 |
| <7 | 5,274 | (6.52%) | 23,189 | (1.59%) | 0.252 | 4,324 | (5.92%) | 4,265 | (5.84%) | 0.003 |
| 7-10 | 14,587 | (18.03%) | 62,805 | (4.30%) | 0.447 | 12,055 | (16.50%) | 12,170 | (16.66%) | 0.004 |
| ≥10 | 42,073 | (52.00%) | 219,075 | (15.00%) | 0.852 | 35,946 | (49.20%) | 37,002 | (50.64%) | 0.029 |
| Persons with potential health hazards related to family and personal history and certain conditions influencing health status | 6,114 | (7.56%) | 27,767 | (1.90%) | 0.269 | 4,791 | (6.56%) | 4,651 | (6.37%) | 0.008 |
| Tobacco use | 4,031 | (4.98%) | 20,934 | (1.43%) | 0.202 | 3,303 | (4.52%) | 3,370 | (4.61%) | 0.004 |
| Alcohol related disorders | 6,380 | (7.89%) | 39,064 | (2.67%) | 0.235 | 5,358 | (7.33%) | 5,388 | (7.37%) | 0.002 |
| Diseases of the circulatory system | 67,596 | (83.54%) | 514,449 | (35.22%) | 1.130 | 59,921 | (82.01%) | 61,765 | (84.54%) | 0.068 |
| Hypertensive diseases | 55,379 | (68.44%) | 362,141 | (24.79%) | 0.973 | 48,441 | (66.30%) | 49,632 | (67.93%) | 0.035 |
| Ischemic heart diseases | 22,955 | (28.37%) | 102,001 | (6.98%) | 0.584 | 19,032 | (26.05%) | 18,970 | (25.96%) | 0.002 |
| Diseases of arteries, arterioles and capillaries | 20,631 | (25.50%) | 103,521 | (7.09%) | 0.515 | 17,250 | (23.61%) | 17,235 | (23.59%) | 0.000 |
| Disorders of lipoprotein metabolism and other lipidemias | 57,627 | (71.22%) | 367,020 | (25.13%) | 1.040 | 50,572 | (69.22%) | 52,288 | (71.57%) | 0.051 |
| Chronic lower respiratory diseases | 36,411 | (45.00%) | 199,971 | (13.69%) | 0.732 | 30,643 | (41.94%) | 30,709 | (42.03%) | 0.002 |
| Pulmonary heart disease and diseases of pulmonary circulation | 9,852 | (12.18%) | 24,101 | (1.65%) | 0.424 | 7,292 | (9.98%) | 6,776 | (9.27%) | 0.024 |
| Diabetes mellitus | 29,363 | (36.29%) | 189,149 | (12.95%) | 0.563 | 24,761 | (33.89%) | 24,651 | (33.74%) | 0.003 |
| Cerebrovascular diseases | 13,685 | (16.91%) | 75,390 | (5.16%) | 0.382 | 11,590 | (15.86%) | 11,597 | (15.87%) | 0.000 |
| Acute kidney failure and chronic kidney disease | 15,777 | (19.50%) | 68,381 | (4.68%) | 0.467 | 12,763 | (17.47%) | 12,588 | (17.23%) | 0.006 |
| Primary thrombophilia | 1,571 | (1.94%) | 6,852 | (0.47%) | 0.135 | 1,266 | (1.73%) | 1,197 | (1.64%) | 0.007 |
| Other thrombophilia | 1,355 | (1.67%) | 5,687 | (0.39%) | 0.127 | 1,078 | (1.48%) | 1,075 | (1.47%) | 0.000 |
| Polycythemia vera | 713 | (0.88%) | 1,672 | (0.11%) | 0.109 | 520 | (0.71%) | 480 | (0.66%) | 0.007 |
| Homocystinuria | 331 | (0.41%) | 1,267 | (0.09%) | 0.065 | 269 | (0.37%) | 254 | (0.35%) | 0.003 |
| Malignant immunoproliferative diseases and certain other B-cell lymphomas | 91 | (0.11%) | 893 | (0.06%) | 0.017 | 83 | (0.11%) | 89 | (0.12%) | 0.002 |
| Multiple myeloma and malignant plasma cell neoplasms | 378 | (0.47%) | 2,845 | (0.19%) | 0.047 | 316 | (0.43%) | 320 | (0.44%) | 0.001 |
| Insomnia | 23,544 | (29.10%) | 67,256 | (4.60%) | 0.692 | 18,666 | (25.55%) | 18,551 | (25.39%) | 0.004 |
| Circadian rhythm sleep disorders | 2,667 | (3.30%) | 1,780 | (0.12%) | 0.247 | 1,303 | (1.78%) | 1,047 | (1.43%) | 0.028 |
| Sleep disorders not due to a substance or known physiological condition | 9,552 | (11.81%) | 18,035 | (1.23%) | 0.438 | 6,926 | (9.48%) | 6,541 | (8.95%) | 0.018 |
| Glaucoma | 13,348 | (16.50%) | 181,420 | (12.42%) | 0.116 | 11,784 | (16.13%) | 11,543 | (15.80%) | 0.009 |
| Type 1 diabetes mellitus with ophthalmic complications | 952 | (1.18%) | 8,251 | (0.56%) | 0.066 | 790 | (1.08%) | 739 | (1.01%) | 0.007 |
| Type 2 diabetes mellitus with ophthalmic complications | 6,041 | (7.47%) | 38,283 | (2.62%) | 0.223 | 4,922 | (6.74%) | 4,730 | (6.47%) | 0.011 |
| Nonexudative age-related macular degeneration | 2,801 | (3.46%) | 44,180 | (3.02%) | 0.025 | 2,532 | (3.47%) | 2,609 | (3.57%) | 0.006 |
| Exudative age-related macular degeneration | 766 | (0.95%) | 19,593 | (1.34%) | 0.037 | 702 | (0.96%) | 697 | (0.95%) | 0.001 |
| Continuous positive airway pressure ventilation therapy | 5,567 | (6.88%) | 2,243 | (0.15%) | 0.371 | 2,318 | (3.17%) | 1,699 | (2.33%) | 0.052 |
| *Medications* |  |  |  |  |  |  |  |  |  |  |
| Antithrombotic agents | 48,762 | (60.26%) | 319,160 | (21.85%) | 0.848 | 42,163 | (57.71%) | 42,916 | (58.74%) | 0.021 |
| Antihypertensives | 14,258 | (17.62%) | 68,752 | (4.71%) | 0.419 | 11,440 | (15.66%) | 11,343 | (15.53%) | 0.004 |
| Lipid modifying agents | 45,000 | (55.62%) | 299,640 | (20.51%) | 0.775 | 39,081 | (53.49%) | 39,862 | (54.56%) | 0.021 |
| Hypnotics and sedatives | 47,589 | (58.82%) | 276,158 | (18.91%) | 0.897 | 40,718 | (55.73%) | 41,659 | (57.02%) | 0.026 |
| Estrogens | 12,640 | (15.62%) | 126,198 | (8.64%) | 0.215 | 11,049 | (15.12%) | 11,250 | (15.40%) | 0.008 |

SMD = standardized mean difference

eTable 4D: Results of the propensity score matching analyses performed on OSA-naïve Hispanic/Latino patients

|  | **Before Matching** | | | | | **After Matching** | | | | |
| --- | --- | --- | --- | --- | --- | --- | --- | --- | --- | --- |
|  | **Obstructive Sleep Apnea** | | **Control** | | **SMD** | **Obstructive Sleep Apnea** | | **Control** | | **SMD** |
| Number of Patients | 16,536 | | 323,125 | |  | 15,449 | | 15,449 | |  |
| Age at Index | 49.61 | (17.72) | 20.75 | (20.75) | 0.353 | 49.32 | (17.89) | 49.88 | (18.61) | 0.031 |
| *Sex* |  |  |  |  |  |  |  |  |  |  |
| Male | 7,398 | (44.74%) | 138,402 | (42.83%) | 0.038 | 6,940 | (44.92%) | 6,998 | (45.30%) | 0.008 |
| Female | 9,138 | (55.26%) | 184,685 | (57.16%) | 0.038 | 8,509 | (55.08%) | 8,448 | (54.68%) | 0.008 |
| *Ethnicity* |  |  |  |  |  |  |  |  |  |  |
| Hispanic or Latino | 16,536 | (100.00%) | 323,125 | (100.00%) |  | 15,449 | (100.00%) | 15,449 | (100.00%) |  |
| Not Hispanic or Latino | 0 | (0.00%) | 0 | (0.00%) |  | 0 | (0.00%) | 0 | (0.00%) |  |
| *Race* |  |  |  |  |  |  |  |  |  |  |
| White | 9,486 | (57.37%) | 168,199 | (52.05%) | 0.107 | 8,920 | (57.74%) | 9,215 | (59.65%) | 0.039 |
| Black or African American | 500 | (3.02%) | 6,206 | (1.92%) | 0.071 | 445 | (2.88%) | 458 | (2.96%) | 0.005 |
| Asian | 46 | (0.28%) | 1,450 | (0.45%) | 0.028 | 46 | (0.30%) | 43 | (0.28%) | 0.004 |
| Native Hawaiian or Other Pacific Islander | 65 | (0.39%) | 934 | (0.29%) | 0.018 | 61 | (0.39%) | 52 | (0.34%) | 0.010 |
| American Indian or Alaska Native | 108 | (0.65%) | 1,734 | (0.54%) | 0.015 | 101 | (0.65%) | 99 | (0.64%) | 0.002 |
| Other known race (unspecified) | 2,842 | (17.19%) | 79,073 | (24.47%) | 0.180 | 2,636 | (17.06%) | 2,472 | (16.00%) | 0.029 |
| *Body Mass Index (kg/m^2^)* | 9,249 | (55.93%) | 129,178 | (39.98%) | 0.894 | 8,321 | (53.86%) | 8,607 | (55.71%) | 0.272 |
| <18.5 | 628 | (3.80%) | 12,498 | (3.87%) | 0.004 | 572 | (3.70%) | 539 | (3.49%) | 0.011 |
| 18.5-24.9 | 2,065 | (12.49%) | 49,609 | (15.35%) | 0.083 | 1,941 | (12.56%) | 1,972 | (12.76%) | 0.006 |
| 25-29.9 | 4,155 | (25.13%) | 60,782 | (18.81%) | 0.153 | 3,836 | (24.83%) | 4,002 | (25.90%) | 0.025 |
| 30-34.9 | 5,059 | (30.59%) | 41,646 | (12.89%) | 0.439 | 4,481 | (29.01%) | 4,436 | (28.71%) | 0.006 |
| 35-40 | 4,092 | (24.75%) | 19,187 | (5.94%) | 0.541 | 3,458 | (22.38%) | 3,247 | (21.02%) | 0.033 |
| >40.0 | 3,300 | (19.96%) | 10,167 | (3.15%) | 0.545 | 2,682 | (17.36%) | 2,519 | (16.31%) | 0.028 |
| *Hemoglobin A1c (%)* | 10,055 | (60.81%) | 81,587 | (25.25%) | 0.090 | 9,127 | (59.08%) | 9,603 | (62.16%) | 0.029 |
| <7 | 2,095 | (12.67%) | 16,941 | (5.24%) | 0.262 | 1,884 | (12.19%) | 1,982 | (12.83%) | 0.019 |
| 7-10 | 4,161 | (25.16%) | 27,796 | (8.60%) | 0.453 | 3,733 | (24.16%) | 3,877 | (25.10%) | 0.022 |
| ≥10 | 8,630 | (52.19%) | 62,430 | (19.32%) | 0.730 | 7,781 | (50.37%) | 8,115 | (52.53%) | 0.043 |
| Persons with potential health hazards related to family and personal history and certain conditions influencing health status | 1,601 | (9.68%) | 10,700 | (3.31%) | 0.261 | 1,355 | (8.77%) | 1,328 | (8.60%) | 0.006 |
| Tobacco use | 766 | (4.63%) | 4,887 | (1.51%) | 0.182 | 669 | (4.33%) | 706 | (4.57%) | 0.012 |
| Alcohol related disorders | 1,030 | (6.23%) | 7,639 | (2.36%) | 0.191 | 900 | (5.83%) | 876 | (5.67%) | 0.007 |
| Diseases of the circulatory system | 13,022 | (78.75%) | 111,466 | (34.50%) | 0.998 | 11,964 | (77.44%) | 12,468 | (80.70%) | 0.080 |
| Hypertensive diseases | 10,974 | (66.36%) | 88,302 | (27.33%) | 0.850 | 10,010 | (64.79%) | 10,360 | (67.06%) | 0.048 |
| Ischemic heart diseases | 3,924 | (23.73%) | 16,318 | (5.05%) | 0.552 | 3,401 | (22.01%) | 3,434 | (22.23%) | 0.005 |
| Diseases of arteries, arterioles and capillaries | 3,162 | (19.12%) | 15,273 | (4.73%) | 0.456 | 2,733 | (17.69%) | 2,729 | (17.66%) | 0.001 |
| Disorders of lipoprotein metabolism and other lipidemias | 10,395 | (62.86%) | 75,880 | (23.48%) | 0.866 | 9,437 | (61.08%) | 9,738 | (63.03%) | 0.040 |
| Chronic lower respiratory diseases | 6,135 | (37.10%) | 31,150 | (9.64%) | 0.686 | 5,328 | (34.49%) | 5,224 | (33.81%) | 0.014 |
| Pulmonary heart disease and diseases of pulmonary circulation | 1,405 | (8.50%) | 3,558 | (1.10%) | 0.351 | 1,099 | (7.11%) | 1,012 | (6.55%) | 0.022 |
| Diabetes mellitus | 8,526 | (51.56%) | 92,240 | (28.55%) | 0.483 | 7,784 | (50.39%) | 7,932 | (51.34%) | 0.019 |
| Cerebrovascular diseases | 2,462 | (14.89%) | 13,891 | (4.30%) | 0.366 | 2,158 | (13.97%) | 2,111 | (13.66%) | 0.009 |
| Acute kidney failure and chronic kidney disease | 2,777 | (16.79%) | 17,080 | (5.29%) | 0.374 | 2,414 | (15.63%) | 2,483 | (16.07%) | 0.012 |
| Primary thrombophilia | 156 | (0.94%) | 763 | (0.24%) | 0.092 | 126 | (0.82%) | 135 | (0.87%) | 0.006 |
| Other thrombophilia | 144 | (0.87%) | 846 | (0.26%) | 0.081 | 118 | (0.76%) | 110 | (0.71%) | 0.006 |
| Polycythemia vera | 83 | (0.50%) | 161 | (0.05%) | 0.086 | 63 | (0.41%) | 58 | (0.38%) | 0.005 |
| Homocystinuria | 32 | (0.19%) | 121 | (0.04%) | 0.046 | 27 | (0.17%) | 27 | (0.17%) | 0.000 |
| Malignant immunoproliferative diseases and certain other B-cell lymphomas | 14 | (0.08%) | 104 | (0.03%) | 0.022 | 12 | (0.08%) | 10 | (0.06%) | 0.005 |
| Multiple myeloma and malignant plasma cell neoplasms | 37 | (0.22%) | 461 | (0.14%) | 0.019 | 32 | (0.21%) | 27 | (0.17%) | 0.007 |
| Insomnia | 4,102 | (24.81%) | 11,648 | (3.60%) | 0.637 | 3,350 | (21.68%) | 3,267 | (21.15%) | 0.013 |
| Circadian rhythm sleep disorders | 401 | (2.43%) | 231 | (0.07%) | 0.213 | 203 | (1.31%) | 164 | (1.06%) | 0.023 |
| Sleep disorders not due to a substance or known physiological condition | 1,585 | (9.59%) | 3,588 | (1.11%) | 0.384 | 1,233 | (7.98%) | 1,158 | (7.50%) | 0.018 |
| Glaucoma | 3,174 | (19.19%) | 40,958 | (12.68%) | 0.179 | 2,873 | (18.60%) | 2,885 | (18.67%) | 0.002 |
| Type 1 diabetes mellitus with ophthalmic complications | 209 | (1.26%) | 1,755 | (0.54%) | 0.076 | 177 | (1.15%) | 180 | (1.17%) | 0.002 |
| Type 2 diabetes mellitus with ophthalmic complications | 2,482 | (15.01%) | 29,164 | (9.03%) | 0.185 | 2,247 | (14.54%) | 2,218 | (14.36%) | 0.005 |
| Nonexudative age-related macular degeneration | 206 | (1.25%) | 2,481 | (0.77%) | 0.048 | 190 | (1.23%) | 187 | (1.21%) | 0.002 |
| Exudative age-related macular degeneration | 64 | (0.39%) | 844 | (0.26%) | 0.022 | 56 | (0.36%) | 52 | (0.34%) | 0.004 |
| Continuous positive airway pressure ventilation therapy | 635 | (3.84%) | 449 | (0.14%) | 0.267 | 360 | (2.33%) | 269 | (1.74%) | 0.042 |
| *Medications* |  |  |  |  |  |  |  |  |  |  |
| Antithrombotic agents | 7,607 | (46.00%) | 48,949 | (15.15%) | 0.711 | 6,782 | (43.90%) | 6,828 | (44.20%) | 0.006 |
| Antihypertensives | 2,472 | (14.95%) | 12,668 | (3.92%) | 0.384 | 2,093 | (13.55%) | 2,083 | (13.48%) | 0.002 |
| Lipid modifying agents | 8,146 | (49.26%) | 60,277 | (18.65%) | 0.683 | 7,366 | (47.68%) | 7,518 | (48.66%) | 0.020 |
| Hypnotics and sedatives | 7,139 | (43.17%) | 40,620 | (12.57%) | 0.726 | 6,218 | (40.25%) | 6,239 | (40.38%) | 0.003 |
| Estrogens | 2,181 | (13.19%) | 17,550 | (5.43%) | 0.269 | 1,914 | (12.39%) | 1,845 | (11.94%) | 0.014 |

SMD = standardized mean difference

eTable 4E: Results of the propensity score matching analyses performed on OSA-naïve Black patients

|  | **Before Matching** | | | | | **After Matching** | | | | |
| --- | --- | --- | --- | --- | --- | --- | --- | --- | --- | --- |
|  | **Obstructive Sleep Apnea** | | **Control** | | **SMD** | **Obstructive Sleep Apnea** | | **Control** | | **SMD** |
| Number of Patients | 34,158 | | 423,178 | |  | 28,899 | | 28,899 | |  |
| Age at Index | 52.58 | (15.73) | 20.69 | (20.69) | 0.304 | 52.19 | (16.10) | 52.55 | (16.94) | 0.022 |
| *Sex* |  |  |  |  |  |  |  |  |  |  |
| Male | 11,831 | (34.64%) | 171,400 | (40.50%) | 0.121 | 10,357 | (35.84%) | 10,145 | (35.11%) | 0.015 |
| Female | 22,325 | (65.36%) | 251,714 | (59.48%) | 0.122 | 18,540 | (64.15%) | 18,752 | (64.89%) | 0.015 |
| *Ethnicity* |  |  |  |  |  |  |  |  |  |  |
| Hispanic or Latino | 499 | (1.46%) | 6,201 | (1.47%) | 0.000 | 437 | (1.51%) | 465 | (1.61%) | 0.008 |
| Not Hispanic or Latino | 32,940 | (96.43%) | 387,070 | (91.47%) | 0.209 | 27,786 | (96.15%) | 27,727 | (95.94%) | 0.010 |
| *Race* |  |  |  |  |  |  |  |  |  |  |
| White | 0 | (0.00%) | 0 | (0.00%) |  | 0 | (0.00%) | 0 | (0.00%) |  |
| Black or African American | 34,158 | (100.00%) | 423,178 | (100.00%) |  | 28,899 | (100.00%) | 28,899 | (100.00%) |  |
| Asian | 0 | (0.00%) | 0 | (0.00%) |  | 0 | (0.00%) | 0 | (0.00%) |  |
| Native Hawaiian or Other Pacific Islander | 0 | (0.00%) | 0 | (0.00%) |  | 0 | (0.00%) | 0 | (0.00%) |  |
| American Indian or Alaska Native | 0 | (0.00%) | 0 | (0.00%) |  | 0 | (0.00%) | 0 | (0.00%) |  |
| Other known race (unspecified) | 0 | (0.00%) | 0 | (0.00%) |  | 0 | (0.00%) | 0 | (0.00%) |  |
| *Body Mass Index (kg/m^2^)* | 26,635 | (77.98%) | 215,335 | (50.89%) | 1.019 | 21,889 | (75.74%) | 21,859 | (75.64%) | 0.310 |
| <18.5 | 2,352 | (6.89%) | 24,689 | (5.83%) | 0.043 | 1,890 | (6.54%) | 1,856 | (6.42%) | 0.005 |
| 18.5-24.9 | 5,137 | (15.04%) | 84,381 | (19.94%) | 0.129 | 4,456 | (15.42%) | 4,552 | (15.75%) | 0.009 |
| 25-29.9 | 9,631 | (28.20%) | 98,401 | (23.25%) | 0.113 | 8,314 | (28.77%) | 8,750 | (30.28%) | 0.033 |
| 30-34.9 | 13,507 | (39.54%) | 75,897 | (17.94%) | 0.492 | 11,007 | (38.09%) | 11,635 | (40.26%) | 0.045 |
| 35-40 | 13,299 | (38.93%) | 42,372 | (10.01%) | 0.714 | 10,057 | (34.80%) | 10,359 | (35.85%) | 0.022 |
| >40.0 | 13,642 | (39.94%) | 27,293 | (6.45%) | 0.864 | 9,777 | (33.83%) | 9,740 | (33.70%) | 0.003 |
| *Hemoglobin A1c (%)* | 21,841 | (63.94%) | 110,069 | (26.01%) | 0.041 | 17,425 | (60.30%) | 17,794 | (61.57%) | 0.021 |
| <7 | 3,966 | (11.61%) | 18,659 | (4.41%) | 0.268 | 3,090 | (10.69%) | 3,095 | (10.71%) | 0.001 |
| 7-10 | 8,185 | (23.96%) | 32,580 | (7.70%) | 0.457 | 6,278 | (21.72%) | 6,269 | (21.69%) | 0.001 |
| ≥10 | 18,989 | (55.59%) | 89,923 | (21.25%) | 0.755 | 14,981 | (51.84%) | 15,444 | (53.44%) | 0.032 |
| Persons with potential health hazards related to family and personal history and certain conditions influencing health status | 3,732 | (10.93%) | 17,119 | (4.05%) | 0.264 | 2,733 | (9.46%) | 2,788 | (9.65%) | 0.006 |
| Tobacco use | 3,229 | (9.45%) | 11,798 | (2.79%) | 0.281 | 2,427 | (8.40%) | 2,408 | (8.33%) | 0.002 |
| Alcohol related disorders | 2,731 | (8.00%) | 15,992 | (3.78%) | 0.180 | 2,170 | (7.51%) | 2,173 | (7.52%) | 0.000 |
| Diseases of the circulatory system | 29,754 | (87.11%) | 187,756 | (44.37%) | 1.009 | 24,596 | (85.11%) | 25,261 | (87.41%) | 0.067 |
| Hypertensive diseases | 27,393 | (80.19%) | 162,435 | (38.38%) | 0.940 | 22,447 | (77.67%) | 22,944 | (79.39%) | 0.042 |
| Ischemic heart diseases | 9,030 | (26.44%) | 28,689 | (6.78%) | 0.548 | 6,608 | (22.87%) | 6,540 | (22.63%) | 0.006 |
| Diseases of arteries, arterioles and capillaries | 6,492 | (19.01%) | 23,461 | (5.54%) | 0.419 | 4,865 | (16.83%) | 4,915 | (17.01%) | 0.005 |
| Disorders of lipoprotein metabolism and other lipidemias | 22,176 | (64.92%) | 107,069 | (25.30%) | 0.868 | 17,746 | (61.41%) | 17,985 | (62.23%) | 0.017 |
| Chronic lower respiratory diseases | 16,115 | (47.18%) | 62,227 | (14.70%) | 0.750 | 12,114 | (41.92%) | 12,086 | (41.82%) | 0.002 |
| Pulmonary heart disease and diseases of pulmonary circulation | 4,951 | (14.49%) | 9,571 | (2.26%) | 0.453 | 3,275 | (11.33%) | 3,172 | (10.98%) | 0.011 |
| Diabetes mellitus | 18,152 | (53.14%) | 110,428 | (26.09%) | 0.575 | 14,478 | (50.10%) | 14,569 | (50.41%) | 0.006 |
| Cerebrovascular diseases | 5,488 | (16.07%) | 25,060 | (5.92%) | 0.329 | 4,194 | (14.51%) | 4,299 | (14.88%) | 0.010 |
| Acute kidney failure and chronic kidney disease | 9,336 | (27.33%) | 38,072 | (9.00%) | 0.490 | 7,001 | (24.23%) | 7,086 | (24.52%) | 0.007 |
| Primary thrombophilia | 387 | (1.13%) | 1,226 | (0.29%) | 0.100 | 269 | (0.93%) | 280 | (0.97%) | 0.004 |
| Other thrombophilia | 315 | (0.92%) | 1,141 | (0.27%) | 0.085 | 226 | (0.78%) | 226 | (0.78%) | 0.000 |
| Polycythemia vera | 111 | (0.32%) | 248 | (0.06%) | 0.061 | 80 | (0.28%) | 74 | (0.26%) | 0.004 |
| Homocystinuria | 59 | (0.17%) | 217 | (0.05%) | 0.036 | 39 | (0.13%) | 30 | (0.10%) | 0.009 |
| Malignant immunoproliferative diseases and certain other B-cell lymphomas | 28 | (0.08%) | 157 | (0.04%) | 0.018 | 19 | (0.07%) | 25 | (0.09%) | 0.008 |
| Multiple myeloma and malignant plasma cell neoplasms | 194 | (0.57%) | 1,241 | (0.29%) | 0.042 | 155 | (0.54%) | 161 | (0.56%) | 0.003 |
| Insomnia | 8,667 | (25.37%) | 16,194 | (3.83%) | 0.641 | 5,828 | (20.17%) | 5,751 | (19.90%) | 0.007 |
| Circadian rhythm sleep disorders | 1,172 | (3.43%) | 382 | (0.09%) | 0.256 | 386 | (1.34%) | 296 | (1.02%) | 0.029 |
| Sleep disorders not due to a substance or known physiological condition | 3,397 | (9.94%) | 3,979 | (0.94%) | 0.405 | 1,994 | (6.90%) | 1,869 | (6.47%) | 0.017 |
| Glaucoma | 8,873 | (25.98%) | 83,890 | (19.82%) | 0.147 | 7,250 | (25.09%) | 6,940 | (24.01%) | 0.025 |
| Type 1 diabetes mellitus with ophthalmic complications | 487 | (1.43%) | 2,577 | (0.61%) | 0.081 | 381 | (1.32%) | 360 | (1.25%) | 0.006 |
| Type 2 diabetes mellitus with ophthalmic complications | 4,579 | (13.41%) | 27,636 | (6.53%) | 0.231 | 3,589 | (12.42%) | 3,460 | (11.97%) | 0.014 |
| Nonexudative age-related macular degeneration | 231 | (0.68%) | 1,970 | (0.47%) | 0.028 | 178 | (0.62%) | 184 | (0.64%) | 0.003 |
| Exudative age-related macular degeneration | 80 | (0.23%) | 660 | (0.16%) | 0.018 | 61 | (0.21%) | 63 | (0.22%) | 0.001 |
| Continuous positive airway pressure ventilation therapy | 2,565 | (7.51%) | 1,120 | (0.26%) | 0.382 | 1,019 | (3.53%) | 784 | (2.71%) | 0.047 |
| *Medications* |  |  |  |  |  |  |  |  |  |  |
| Antithrombotic agents | 20,165 | (59.03%) | 95,555 | (22.58%) | 0.799 | 15,837 | (54.80%) | 16,031 | (55.47%) | 0.013 |
| Antihypertensives | 9,452 | (27.67%) | 34,529 | (8.16%) | 0.526 | 7,023 | (24.30%) | 7,036 | (24.35%) | 0.001 |
| Lipid modifying agents | 18,971 | (55.54%) | 93,176 | (22.02%) | 0.733 | 15,034 | (52.02%) | 15,235 | (52.72%) | 0.014 |
| Hypnotics and sedatives | 20,296 | (59.42%) | 69,848 | (16.51%) | 0.986 | 15,615 | (54.03%) | 15,799 | (54.67%) | 0.013 |
| Estrogens | 4,776 | (13.98%) | 24,604 | (5.81%) | 0.276 | 3,608 | (12.48%) | 3,669 | (12.70%) | 0.006 |

SMD = standardized mean difference

eTable 4F: Results of the propensity score matching analyses performed on OSA-naïve Asian patients

|  | **Before Matching** | | | | | **After Matching** | | | | |
| --- | --- | --- | --- | --- | --- | --- | --- | --- | --- | --- |
|  | **Obstructive Sleep Apnea** | | **Control** | | **SMD** | **Obstructive Sleep Apnea** | | **Control** | | **SMD** |
| Number of Patients | 3,526 | | 137,145 | |  | 3,430 | | 3,430 | |  |
| Age at Index | 54.99 | (17.55) | 20.56 | (20.56) | 0.405 | 54.89 | (17.56) | 55.37 | (17.51) | 0.027 |
| *Sex* |  |  |  |  |  |  |  |  |  |  |
| Male | 2,021 | (57.32%) | 55,431 | (40.42%) | 0.343 | 1,961 | (57.17%) | 1,977 | (57.64%) | 0.009 |
| Female | 1,505 | (42.68%) | 81,671 | (59.55%) | 0.342 | 1,469 | (42.83%) | 1,452 | (42.33%) | 0.010 |
| *Ethnicity* |  |  |  |  |  |  |  |  |  |  |
| Hispanic or Latino | 46 | (1.30%) | 1,450 | (1.06%) | 0.023 | 46 | (1.34%) | 40 | (1.17%) | 0.016 |
| Not Hispanic or Latino | 3,256 | (92.34%) | 117,079 | (85.37%) | 0.223 | 3,164 | (92.24%) | 3,169 | (92.39%) | 0.005 |
| *Race* |  |  |  |  |  |  |  |  |  |  |
| White | 0 | (0.00%) | 0 | (0.00%) |  | 0 | (0.00%) | 0 | (0.00%) |  |
| Black or African American | 0 | (0.00%) | 0 | (0.00%) |  | 0 | (0.00%) | 0 | (0.00%) |  |
| Asian | 3,526 | (100.00%) | 137,145 | (100.00%) |  | 3,430 | (100.00%) | 3,430 | (100.00%) |  |
| Native Hawaiian or Other Pacific Islander | 0 | (0.00%) | 0 | (0.00%) |  | 0 | (0.00%) | 0 | (0.00%) |  |
| American Indian or Alaska Native | 0 | (0.00%) | 0 | (0.00%) |  | 0 | (0.00%) | 0 | (0.00%) |  |
| Other known race (unspecified) | 0 | (0.00%) | 0 | (0.00%) |  | 0 | (0.00%) | 0 | (0.00%) |  |
| *Body Mass Index (kg/m^2^)* | 2,608 | (73.96%) | 61,000 | (44.48%) | 0.747 | 2,518 | (73.41%) | 2,546 | (74.23%) | 0.177 |
| <18.5 | 132 | (3.74%) | 7,408 | (5.40%) | 0.079 | 129 | (3.76%) | 122 | (3.56%) | 0.011 |
| 18.5-24.9 | 1,218 | (34.54%) | 38,347 | (27.96%) | 0.142 | 1,178 | (34.34%) | 1,248 | (36.38%) | 0.043 |
| 25-29.9 | 1,590 | (45.09%) | 26,226 | (19.12%) | 0.579 | 1,531 | (44.64%) | 1,621 | (47.26%) | 0.053 |
| 30-34.9 | 992 | (28.13%) | 8,710 | (6.35%) | 0.602 | 939 | (27.38%) | 967 | (28.19%) | 0.018 |
| 35-40 | 481 | (13.64%) | 2,382 | (1.74%) | 0.458 | 441 | (12.86%) | 402 | (11.72%) | 0.035 |
| >40.0 | 313 | (8.88%) | 1,146 | (0.84%) | 0.381 | 278 | (8.10%) | 249 | (7.26%) | 0.032 |
| *Hemoglobin A1c (%)* | 1,686 | (47.82%) | 26,941 | (19.64%) | 0.054 | 1,617 | (47.14%) | 1,704 | (49.68%) | 0.007 |
| <7 | 151 | (4.28%) | 2,064 | (1.50%) | 0.166 | 146 | (4.26%) | 140 | (4.08%) | 0.009 |
| 7-10 | 532 | (15.09%) | 6,960 | (5.07%) | 0.337 | 510 | (14.87%) | 522 | (15.22%) | 0.010 |
| ≥10 | 1,517 | (43.02%) | 23,349 | (17.03%) | 0.591 | 1,453 | (42.36%) | 1,539 | (44.87%) | 0.051 |
| Persons with potential health hazards related to family and personal history and certain conditions influencing health status | 198 | (5.62%) | 2,423 | (1.77%) | 0.205 | 185 | (5.39%) | 196 | (5.71%) | 0.014 |
| Tobacco use | 68 | (1.93%) | 792 | (0.58%) | 0.122 | 63 | (1.84%) | 60 | (1.75%) | 0.007 |
| Alcohol related disorders | 56 | (1.59%) | 791 | (0.58%) | 0.098 | 50 | (1.46%) | 56 | (1.63%) | 0.014 |
| Diseases of the circulatory system | 2,616 | (74.19%) | 39,533 | (28.83%) | 1.019 | 2,526 | (73.64%) | 2,676 | (78.02%) | 0.102 |
| Hypertensive diseases | 2,169 | (61.51%) | 30,794 | (22.45%) | 0.862 | 2,090 | (60.93%) | 2,199 | (64.11%) | 0.066 |
| Ischemic heart diseases | 739 | (20.96%) | 6,391 | (4.66%) | 0.503 | 694 | (20.23%) | 685 | (19.97%) | 0.007 |
| Diseases of arteries, arterioles and capillaries | 610 | (17.30%) | 5,100 | (3.72%) | 0.454 | 570 | (16.62%) | 581 | (16.94%) | 0.009 |
| Disorders of lipoprotein metabolism and other lipidemias | 2,378 | (67.44%) | 33,713 | (24.58%) | 0.952 | 2,294 | (66.88%) | 2,395 | (69.83%) | 0.063 |
| Chronic lower respiratory diseases | 1,004 | (28.47%) | 9,847 | (7.18%) | 0.579 | 942 | (27.46%) | 903 | (26.33%) | 0.026 |
| Pulmonary heart disease and diseases of pulmonary circulation | 208 | (5.90%) | 1,078 | (0.79%) | 0.287 | 188 | (5.48%) | 179 | (5.22%) | 0.012 |
| Diabetes mellitus | 1,537 | (43.59%) | 26,539 | (19.35%) | 0.541 | 1,474 | (42.97%) | 1,488 | (43.38%) | 0.008 |
| Cerebrovascular diseases | 534 | (15.14%) | 4,697 | (3.42%) | 0.412 | 500 | (14.58%) | 505 | (14.72%) | 0.004 |
| Acute kidney failure and chronic kidney disease | 491 | (13.93%) | 5,100 | (3.72%) | 0.366 | 458 | (13.35%) | 502 | (14.64%) | 0.037 |
| Primary thrombophilia | 14 | (0.40%) | 204 | (0.15%) | 0.048 | 14 | (0.41%) | 20 | (0.58%) | 0.025 |
| Other thrombophilia | 16 | (0.45%) | 247 | (0.18%) | 0.049 | 16 | (0.47%) | 23 | (0.67%) | 0.027 |
| Polycythemia vera | 28 | (0.79%) | 80 | (0.06%) | 0.113 | 25 | (0.73%) | 21 | (0.61%) | 0.014 |
| Homocystinuria | 10 | (0.28%) | 44 | (0.03%) | 0.063 | 10 | (0.29%) | 12 | (0.35%) | 0.010 |
| Malignant immunoproliferative diseases and certain other B-cell lymphomas | 10 | (0.28%) | 49 | (0.04%) | 0.062 | 10 | (0.29%) | 10 | (0.29%) | 0.000 |
| Multiple myeloma and malignant plasma cell neoplasms | 10 | (0.28%) | 145 | (0.11%) | 0.040 | 10 | (0.29%) | 11 | (0.32%) | 0.005 |
| Insomnia | 764 | (21.67%) | 3,947 | (2.88%) | 0.598 | 701 | (20.44%) | 675 | (19.68%) | 0.019 |
| Circadian rhythm sleep disorders | 138 | (3.91%) | 131 | (0.10%) | 0.275 | 102 | (2.97%) | 70 | (2.04%) | 0.060 |
| Sleep disorders not due to a substance or known physiological condition | 402 | (11.40%) | 1,264 | (0.92%) | 0.447 | 363 | (10.58%) | 336 | (9.80%) | 0.026 |
| Glaucoma | 816 | (23.14%) | 22,628 | (16.50%) | 0.167 | 786 | (22.92%) | 794 | (23.15%) | 0.006 |
| Type 1 diabetes mellitus with ophthalmic complications | 23 | (0.65%) | 321 | (0.23%) | 0.063 | 21 | (0.61%) | 22 | (0.64%) | 0.004 |
| Type 2 diabetes mellitus with ophthalmic complications | 327 | (9.27%) | 5,403 | (3.94%) | 0.216 | 308 | (8.98%) | 296 | (8.63%) | 0.012 |
| Nonexudative age-related macular degeneration | 76 | (2.16%) | 2,253 | (1.64%) | 0.038 | 70 | (2.04%) | 62 | (1.81%) | 0.017 |
| Exudative age-related macular degeneration | 16 | (0.45%) | 680 | (0.50%) | 0.006 | 15 | (0.44%) | 10 | (0.29%) | 0.024 |
| Continuous positive airway pressure ventilation therapy | 88 | (2.50%) | 119 | (0.09%) | 0.215 | 65 | (1.90%) | 51 | (1.49%) | 0.032 |
| *Medications* |  |  |  |  |  |  |  |  |  |  |
| Antithrombotic agents | 1,531 | (43.42%) | 19,012 | (13.86%) | 0.692 | 1,454 | (42.39%) | 1,484 | (43.27%) | 0.018 |
| Antihypertensives | 478 | (13.56%) | 4,289 | (3.13%) | 0.384 | 436 | (12.71%) | 418 | (12.19%) | 0.016 |
| Lipid modifying agents | 1,843 | (52.27%) | 26,536 | (19.35%) | 0.731 | 1,769 | (51.57%) | 1,810 | (52.77%) | 0.024 |
| Hypnotics and sedatives | 1,723 | (48.87%) | 17,272 | (12.59%) | 0.855 | 1,640 | (47.81%) | 1,644 | (47.93%) | 0.002 |
| Estrogens | 348 | (9.87%) | 8,051 | (5.87%) | 0.149 | 332 | (9.68%) | 346 | (10.09%) | 0.014 |

SMD = standardized mean difference

eTable 4G: Results of the propensity score matching analyses performed on patients with a previous history of any retinal vein occlusion

|  | **Before Matching** | | | | | **After Matching** | | | | |
| --- | --- | --- | --- | --- | --- | --- | --- | --- | --- | --- |
|  | **Obstructive Sleep Apnea** | | **Control** | | **SMD** | **Obstructive Sleep Apnea** | | **Control** | | **SMD** |
| Number of Patients | 3,303 | | 14,292 | |  | 2,632 | | 2,632 | |  |
| Age at Index | 67.37 | (12.60) | 15.18 | (15.18) | 0.087 | 67.76 | (12.68) | 67.57 | (14.99) | 0.013 |
| *Sex* |  |  |  |  |  |  |  |  |  |  |
| Male | 1,719 | (52.04%) | 6,312 | (44.16%) | 0.158 | 1,347 | (51.18%) | 1,326 | (50.38%) | 0.016 |
| Female | 1,528 | (46.26%) | 7,790 | (54.51%) | 0.165 | 1,235 | (46.92%) | 1,249 | (47.45%) | 0.011 |
| *Ethnicity* |  |  |  |  |  |  |  |  |  |  |
| Hispanic or Latino | 260 | (7.87%) | 1,130 | (7.91%) | 0.001 | 224 | (8.51%) | 228 | (8.66%) | 0.005 |
| Not Hispanic or Latino | 2,591 | (78.44%) | 10,409 | (72.83%) | 0.131 | 2,030 | (77.13%) | 2,050 | (77.89%) | 0.018 |
| *Race* |  |  |  |  |  |  |  |  |  |  |
| White | 2,161 | (65.43%) | 8,928 | (62.47%) | 0.062 | 1,693 | (64.32%) | 1,728 | (65.65%) | 0.028 |
| Black or African American | 542 | (16.41%) | 1,888 | (13.21%) | 0.090 | 421 | (16.00%) | 406 | (15.43%) | 0.016 |
| Asian | 68 | (2.06%) | 572 | (4.00%) | 0.114 | 64 | (2.43%) | 60 | (2.28%) | 0.010 |
| Native Hawaiian or Other Pacific Islander | 13 | (0.39%) | 34 | (0.24%) | 0.028 | 11 | (0.42%) | 12 | (0.46%) | 0.006 |
| American Indian or Alaska Native | 14 | (0.42%) | 40 | (0.28%) | 0.024 | 10 | (0.38%) | 15 | (0.57%) | 0.028 |
| Other known race (unspecified) | 97 | (2.94%) | 493 | (3.45%) | 0.029 | 76 | (2.89%) | 77 | (2.93%) | 0.002 |
| *Body Mass Index (kg/m^2^)* | 2,651 | (80.26%) | 6,922 | (48.43%) | 0.743 | 2,025 | (76.94%) | 2,016 | (76.60%) | 0.213 |
| <18.5 | 140 | (4.24%) | 507 | (3.55%) | 0.036 | 107 | (4.07%) | 108 | (4.10%) | 0.002 |
| 18.5-24.9 | 663 | (20.07%) | 3,077 | (21.53%) | 0.036 | 579 | (22.00%) | 598 | (22.72%) | 0.017 |
| 25-29.9 | 1,330 | (40.27%) | 3,817 | (26.71%) | 0.290 | 1,076 | (40.88%) | 1,165 | (44.26%) | 0.068 |
| 30-34.9 | 1,423 | (43.08%) | 2,356 | (16.48%) | 0.608 | 1,031 | (39.17%) | 1,063 | (40.39%) | 0.025 |
| 35-40 | 1,060 | (32.09%) | 1,008 | (7.05%) | 0.665 | 632 | (24.01%) | 598 | (22.72%) | 0.031 |
| >40.0 | 804 | (24.34%) | 500 | (3.50%) | 0.631 | 416 | (15.81%) | 356 | (13.53%) | 0.064 |
| *Hemoglobin A1c (%)* | 1,914 | (57.95%) | 3,579 | (25.04%) | 0.061 | 1,375 | (52.24%) | 1,393 | (52.93%) | 0.007 |
| <7 | 350 | (10.60%) | 477 | (3.34%) | 0.288 | 216 | (8.21%) | 224 | (8.51%) | 0.011 |
| 7-10 | 827 | (25.04%) | 1,133 | (7.93%) | 0.474 | 540 | (20.52%) | 532 | (20.21%) | 0.008 |
| ≥10 | 1,679 | (50.83%) | 3,101 | (21.70%) | 0.636 | 1,201 | (45.63%) | 1,224 | (46.50%) | 0.018 |
| Persons with potential health hazards related to family and personal history and certain conditions influencing health status | 180 | (5.45%) | 309 | (2.16%) | 0.172 | 119 | (4.52%) | 122 | (4.64%) | 0.005 |
| Tobacco use | 176 | (5.33%) | 244 | (1.71%) | 0.198 | 118 | (4.48%) | 116 | (4.41%) | 0.004 |
| Alcohol related disorders | 222 | (6.72%) | 400 | (2.80%) | 0.185 | 157 | (5.97%) | 169 | (6.42%) | 0.019 |
| Diseases of the circulatory system | 3,094 | (93.67%) | 8,423 | (58.94%) | 0.895 | 2,424 | (92.10%) | 2,465 | (93.66%) | 0.061 |
| Hypertensive diseases | 2,887 | (87.41%) | 7,206 | (50.42%) | 0.872 | 2,233 | (84.84%) | 2,268 | (86.17%) | 0.038 |
| Ischemic heart diseases | 1,436 | (43.48%) | 2,323 | (16.25%) | 0.623 | 1,004 | (38.15%) | 1,043 | (39.63%) | 0.030 |
| Diseases of arteries, arterioles and capillaries | 1,185 | (35.88%) | 2,139 | (14.97%) | 0.495 | 833 | (31.65%) | 861 | (32.71%) | 0.023 |
| Disorders of lipoprotein metabolism and other lipidemias | 2,594 | (78.53%) | 5,603 | (39.20%) | 0.872 | 1,975 | (75.04%) | 2,047 | (77.77%) | 0.064 |
| Chronic lower respiratory diseases | 1,414 | (42.81%) | 2,192 | (15.34%) | 0.635 | 970 | (36.85%) | 982 | (37.31%) | 0.009 |
| Pulmonary heart disease and diseases of pulmonary circulation | 594 | (17.98%) | 609 | (4.26%) | 0.447 | 355 | (13.49%) | 326 | (12.39%) | 0.033 |
| Diabetes mellitus | 1,752 | (53.04%) | 3,815 | (26.69%) | 0.559 | 1,259 | (47.83%) | 1,237 | (47.00%) | 0.017 |
| Cerebrovascular diseases | 1,056 | (31.97%) | 2,125 | (14.87%) | 0.412 | 770 | (29.26%) | 786 | (29.86%) | 0.013 |
| Acute kidney failure and chronic kidney disease | 1,236 | (37.42%) | 1,979 | (13.85%) | 0.561 | 850 | (32.29%) | 843 | (32.03%) | 0.006 |
| Primary thrombophilia | 88 | (2.66%) | 137 | (0.96%) | 0.128 | 59 | (2.24%) | 60 | (2.28%) | 0.003 |
| Other thrombophilia | 68 | (2.06%) | 131 | (0.92%) | 0.094 | 46 | (1.75%) | 49 | (1.86%) | 0.009 |
| Polycythemia vera | 25 | (0.76%) | 23 | (0.16%) | 0.088 | 17 | (0.65%) | 15 | (0.57%) | 0.010 |
| Homocystinuria | 18 | (0.54%) | 33 | (0.23%) | 0.051 | 14 | (0.53%) | 13 | (0.49%) | 0.005 |
| Malignant immunoproliferative diseases and certain other B-cell lymphomas | 13 | (0.39%) | 28 | (0.20%) | 0.036 | 11 | (0.42%) | 10 | (0.38%) | 0.006 |
| Multiple myeloma and malignant plasma cell neoplasms | 33 | (1.00%) | 68 | (0.48%) | 0.061 | 27 | (1.03%) | 29 | (1.10%) | 0.007 |
| Insomnia | 745 | (22.56%) | 802 | (5.61%) | 0.502 | 468 | (17.78%) | 469 | (17.82%) | 0.001 |
| Circadian rhythm sleep disorders | 58 | (1.76%) | 14 | (0.10%) | 0.174 | 20 | (0.76%) | 14 | (0.53%) | 0.028 |
| Sleep disorders not due to a substance or known physiological condition | 229 | (6.93%) | 208 | (1.46%) | 0.276 | 129 | (4.90%) | 129 | (4.90%) | 0.000 |
| Glaucoma | 1,128 | (34.15%) | 4,714 | (32.98%) | 0.025 | 879 | (33.40%) | 874 | (33.21%) | 0.004 |
| Type 1 diabetes mellitus with ophthalmic complications | 54 | (1.63%) | 89 | (0.62%) | 0.096 | 34 | (1.29%) | 33 | (1.25%) | 0.003 |
| Type 2 diabetes mellitus with ophthalmic complications | 784 | (23.74%) | 1,587 | (11.10%) | 0.338 | 546 | (20.74%) | 535 | (20.33%) | 0.010 |
| Nonexudative age-related macular degeneration | 240 | (7.27%) | 1,067 | (7.47%) | 0.008 | 195 | (7.41%) | 195 | (7.41%) | 0.000 |
| Exudative age-related macular degeneration | 82 | (2.48%) | 451 | (3.16%) | 0.041 | 68 | (2.58%) | 63 | (2.39%) | 0.012 |
| Continuous positive airway pressure ventilation therapy | 277 | (8.39%) | 49 | (0.34%) | 0.402 | 65 | (2.47%) | 48 | (1.82%) | 0.045 |
| *Medications* |  |  |  |  |  |  |  |  |  |  |
| Antithrombotic agents | 2,460 | (74.48%) | 5,763 | (40.32%) | 0.736 | 1,838 | (69.83%) | 1,880 | (71.43%) | 0.035 |
| Antihypertensives | 998 | (30.21%) | 1,678 | (11.74%) | 0.466 | 679 | (25.80%) | 704 | (26.75%) | 0.022 |
| Lipid modifying agents | 2,352 | (71.21%) | 5,403 | (37.80%) | 0.712 | 1,773 | (67.36%) | 1,806 | (68.62%) | 0.027 |
| Hypnotics and sedatives | 2,027 | (61.37%) | 3,629 | (25.39%) | 0.779 | 1,445 | (54.90%) | 1,483 | (56.34%) | 0.029 |
| Estrogens | 330 | (9.99%) | 826 | (5.78%) | 0.157 | 246 | (9.35%) | 269 | (10.22%) | 0.029 |
| *Type of retinal vein occlusion* |  |  |  |  |  |  |  |  |  |  |
| Central retinal vein occlusion | 1,439 | (43.57%) | 6,394 | (44.74%) | 0.024 | 1,140 | (43.31%) | 1,129 | (42.90%) | 0.008 |
| Branch retinal vein occlusion | 2,011 | (60.88%) | 8,228 | (57.57%) | 0.067 | 1,597 | (60.68%) | 1,604 | (60.94%) | 0.005 |

SMD = standardized mean difference

eTable 4H: Results of the propensity score matching analyses performed on patients with a previous history of any retinal vein occlusion but without confounding ocular disorders (sensitivity analysis)

|  | **Before Matching** | | | | | **After Matching** | | | | |
| --- | --- | --- | --- | --- | --- | --- | --- | --- | --- | --- |
|  | **Obstructive Sleep Apnea** | | **Control** | | **SMD** | **Obstructive Sleep Apnea** | | **Control** | | **SMD** |
| Number of Patients | 1,279 | | 14,975 | |  | 896 | | 896 | |  |
| Age at Index | 66.63 | (11.95) | 15.98 | (15.98) | 0.111 | 65.82 | (12.18) | 65.99 | (14.51) | 0.013 |
| *Sex* |  |  |  |  |  |  |  |  |  |  |
| Male | 666 | (52.07%) | 6,277 | (41.92%) | 0.205 | 461 | (51.45%) | 461 | (51.45%) | 0.000 |
| Female | 579 | (45.27%) | 8,156 | (54.46%) | 0.185 | 409 | (45.65%) | 409 | (45.65%) | 0.000 |
| *Ethnicity* |  |  |  |  |  |  |  |  |  |  |
| Hispanic or Latino | 106 | (8.29%) | 898 | (6.00%) | 0.089 | 74 | (8.26%) | 78 | (8.71%) | 0.016 |
| Not Hispanic or Latino | 991 | (77.48%) | 10,163 | (67.87%) | 0.217 | 688 | (76.79%) | 685 | (76.45%) | 0.008 |
| *Race* |  |  |  |  |  |  |  |  |  |  |
| White | 827 | (64.66%) | 9,944 | (66.40%) | 0.037 | 593 | (66.18%) | 616 | (68.75%) | 0.055 |
| Black or African American | 192 | (15.01%) | 1,438 | (9.60%) | 0.165 | 115 | (12.83%) | 100 | (11.16%) | 0.052 |
| Asian | 39 | (3.05%) | 579 | (3.87%) | 0.045 | 29 | (3.24%) | 35 | (3.91%) | 0.036 |
| Native Hawaiian or Other Pacific Islander | 10 | (0.78%) | 174 | (1.16%) | 0.039 | 10 | (1.12%) | 10 | (1.12%) | 0.000 |
| American Indian or Alaska Native | 10 | (0.78%) | 41 | (0.27%) | 0.070 | 10 | (1.12%) | 10 | (1.12%) | 0.000 |
| Other known race (unspecified) | 47 | (3.67%) | 574 | (3.83%) | 0.008 | 39 | (4.35%) | 33 | (3.68%) | 0.034 |
| *Body Mass Index (kg/m^2^)* | 1,038 | (81.16%) | 6,949 | (46.40%) | 0.808 | 698 | (77.90%) | 705 | (78.68%) | 0.293 |
| <18.5 | 55 | (4.30%) | 402 | (2.68%) | 0.088 | 34 | (3.79%) | 37 | (4.13%) | 0.017 |
| 18.5-24.9 | 242 | (18.92%) | 2,947 | (19.68%) | 0.019 | 180 | (20.09%) | 194 | (21.65%) | 0.038 |
| 25-29.9 | 536 | (41.91%) | 3,658 | (24.43%) | 0.378 | 372 | (41.52%) | 417 | (46.54%) | 0.101 |
| 30-34.9 | 580 | (45.35%) | 2,255 | (15.06%) | 0.699 | 369 | (41.18%) | 388 | (43.30%) | 0.043 |
| 35-40 | 458 | (35.81%) | 1,003 | (6.70%) | 0.761 | 249 | (27.79%) | 248 | (27.68%) | 0.002 |
| >40.0 | 343 | (26.82%) | 501 | (3.35%) | 0.694 | 172 | (19.20%) | 162 | (18.08%) | 0.029 |
| *Hemoglobin A1c (%)* | 858 | (67.08%) | 3,721 | (24.85%) | 0.177 | 552 | (61.61%) | 557 | (62.17%) | 0.023 |
| <7 | 170 | (13.29%) | 348 | (2.32%) | 0.418 | 94 | (10.49%) | 91 | (10.16%) | 0.011 |
| 7-10 | 350 | (27.37%) | 861 | (5.75%) | 0.608 | 199 | (22.21%) | 197 | (21.99%) | 0.005 |
| ≥10 | 776 | (60.67%) | 3,329 | (22.23%) | 0.847 | 498 | (55.58%) | 502 | (56.03%) | 0.009 |
| Persons with potential health hazards related to family and personal history and certain conditions influencing health status | 81 | (6.33%) | 258 | (1.72%) | 0.236 | 49 | (5.47%) | 49 | (5.47%) | 0.000 |
| Tobacco use | 66 | (5.16%) | 264 | (1.76%) | 0.187 | 37 | (4.13%) | 38 | (4.24%) | 0.006 |
| Alcohol related disorders | 90 | (7.04%) | 330 | (2.20%) | 0.232 | 54 | (6.03%) | 66 | (7.37%) | 0.054 |
| Diseases of the circulatory system | 1,201 | (93.90%) | 8,729 | (58.29%) | 0.919 | 824 | (91.96%) | 842 | (93.97%) | 0.079 |
| Hypertensive diseases | 1,115 | (87.18%) | 7,189 | (48.01%) | 0.921 | 756 | (84.38%) | 769 | (85.83%) | 0.041 |
| Ischemic heart diseases | 549 | (42.92%) | 2,030 | (13.56%) | 0.690 | 332 | (37.05%) | 351 | (39.17%) | 0.044 |
| Diseases of arteries, arterioles and capillaries | 446 | (34.87%) | 1,827 | (12.20%) | 0.555 | 270 | (30.13%) | 282 | (31.47%) | 0.029 |
| Disorders of lipoprotein metabolism and other lipidemias | 1,054 | (82.41%) | 5,971 | (39.87%) | 0.970 | 704 | (78.57%) | 711 | (79.35%) | 0.019 |
| Chronic lower respiratory diseases | 587 | (45.90%) | 1,939 | (12.95%) | 0.775 | 343 | (38.28%) | 362 | (40.40%) | 0.043 |
| Pulmonary heart disease and diseases of pulmonary circulation | 225 | (17.59%) | 533 | (3.56%) | 0.469 | 108 | (12.05%) | 118 | (13.17%) | 0.034 |
| Diabetes mellitus | 658 | (51.45%) | 2,893 | (19.32%) | 0.713 | 394 | (43.97%) | 379 | (42.30%) | 0.034 |
| Cerebrovascular diseases | 404 | (31.59%) | 1,990 | (13.29%) | 0.450 | 245 | (27.34%) | 258 | (28.79%) | 0.032 |
| Acute kidney failure and chronic kidney disease | 453 | (35.42%) | 1,733 | (11.57%) | 0.586 | 259 | (28.91%) | 274 | (30.58%) | 0.037 |
| Primary thrombophilia | 39 | (3.05%) | 260 | (1.74%) | 0.086 | 26 | (2.90%) | 25 | (2.79%) | 0.007 |
| Other thrombophilia | 36 | (2.81%) | 172 | (1.15%) | 0.120 | 21 | (2.34%) | 20 | (2.23%) | 0.007 |
| Polycythemia vera | 11 | (0.86%) | 30 | (0.20%) | 0.091 | 10 | (1.12%) | 10 | (1.12%) | 0.000 |
| Homocystinuria | 10 | (0.78%) | 42 | (0.28%) | 0.069 | 10 | (1.12%) | 10 | (1.12%) | 0.000 |
| Malignant immunoproliferative diseases and certain other B-cell lymphomas | 10 | (0.78%) | 31 | (0.21%) | 0.082 | 10 | (1.12%) | 10 | (1.12%) | 0.000 |
| Multiple myeloma and malignant plasma cell neoplasms | 16 | (1.25%) | 72 | (0.48%) | 0.083 | 10 | (1.12%) | 10 | (1.12%) | 0.000 |
| Insomnia | 353 | (27.60%) | 739 | (4.93%) | 0.645 | 201 | (22.43%) | 205 | (22.88%) | 0.011 |
| Circadian rhythm sleep disorders | 39 | (3.05%) | 11 | (0.07%) | 0.242 | 12 | (1.34%) | 10 | (1.12%) | 0.020 |
| Sleep disorders not due to a substance or known physiological condition | 143 | (11.18%) | 224 | (1.50%) | 0.406 | 77 | (8.59%) | 73 | (8.15%) | 0.016 |
| Glaucoma | 163 | (12.74%) | 0 | (0.00%) | 0.540 | 10 | (1.12%) | 0 | (0.00%) | 0.150 |
| Type 1 diabetes mellitus with ophthalmic complications | 21 | (1.64%) | 39 | (0.26%) | 0.143 | 11 | (1.23%) | 13 | (1.45%) | 0.019 |
| Type 2 diabetes mellitus with ophthalmic complications | 263 | (20.56%) | 686 | (4.58%) | 0.497 | 135 | (15.07%) | 132 | (14.73%) | 0.009 |
| Nonexudative age-related macular degeneration | 56 | (4.38%) | 428 | (2.86%) | 0.081 | 27 | (3.01%) | 30 | (3.35%) | 0.019 |
| Exudative age-related macular degeneration | 22 | (1.72%) | 0 | (0.00%) | 0.187 | 10 | (1.12%) | 0 | (0.00%) | 0.150 |
| Continuous positive airway pressure ventilation therapy | 160 | (12.51%) | 43 | (0.29%) | 0.516 | 35 | (3.91%) | 32 | (3.57%) | 0.018 |
| *Medications* |  |  |  |  |  |  |  |  |  |  |
| Antithrombotic agents | 884 | (69.12%) | 4,473 | (29.87%) | 0.853 | 572 | (63.84%) | 572 | (63.84%) | 0.000 |
| Antihypertensives | 336 | (26.27%) | 1,224 | (8.17%) | 0.494 | 198 | (22.10%) | 189 | (21.09%) | 0.024 |
| Lipid modifying agents | 866 | (67.71%) | 4,200 | (28.05%) | 0.865 | 564 | (62.95%) | 560 | (62.50%) | 0.009 |
| Hypnotics and sedatives | 733 | (57.31%) | 2,621 | (17.50%) | 0.903 | 461 | (51.45%) | 466 | (52.01%) | 0.011 |
| Estrogens | 127 | (9.93%) | 633 | (4.23%) | 0.224 | 86 | (9.60%) | 84 | (9.38%) | 0.008 |
| *Type of retinal vein occlusion* |  |  |  |  |  |  |  |  |  |  |
| Central retinal vein occlusion | 605 | (47.30%) | 8,351 | (55.77%) | 0.170 | 419 | (46.76%) | 431 | (48.10%) | 0.027 |
| Branch retinal vein occlusion | 719 | (56.22%) | 6,761 | (45.15%) | 0.223 | 500 | (55.80%) | 488 | (54.46%) | 0.027 |

SMD = standardized mean difference
